# Deciphering the Role of Schwann Cells in Inflammatory Peripheral Neuropathies Post Alphavirus Infection

**DOI:** 10.3390/cells12010100

**Published:** 2022-12-26

**Authors:** Yosra Bedoui, Dauriane De Larichaudy, Matthieu Daniel, Franck Ah-Pine, Jimmy Selambarom, Pascale Guiraud, Philippe Gasque

**Affiliations:** 1 Unité de Recherche Etudes Pharmaco-Immunologie (EPI), Université de La Réunion, CHU La Réunion Site Félix Guyon, Allée des Topazes, CS11021, 97400 Saint Denis de La Réunion, France; delarichaudy.dauriane@gmail.com (D.D.L.); matthieu.daniel2309@gmail.com (M.D.); franck.ahpine@gmail.com (F.A.-P.); jimmy.selambarom@univ-reunion.fr (J.S.); pascale.guiraud@univ-reunion.fr (P.G.); philippe.gasque@gmail.com (P.G.); 2 Laboratoire D’immunologie Clinique et Expérimentale de la Zone de L’océan Indien (LICE-OI) CHU La Réunion Site Félix Guyon, Allée des Topazes, CS11021, 97400 Saint Denis de La Réunion, France; 3 Service D’anatomopathologie du CHU Sud de La Réunion, 97410 Saint Pierre, France

**Keywords:** chikungunya, alphavirus, Schwann cells, innate immunity, neuropathology, inflammation

## Abstract

Old world alphaviruses (e.g., chikungunya) are known to cause severe acute and chronic debilitating arthralgia/arthritis. However, atypical neurological manifestations and, in particular, unexpected cases of acute inflammatory Guillain–Barre syndrome (GBS) have been associated with the arthritogenic alphaviruses. The pathogenesis of alphavirus-associated GBS remains unclear. We herein addressed for the first time the role of Schwann cells (SC) in peripheral neuropathy post-alphaviral infection using the prototypical ONNV alphavirus model. We demonstrated that human SC expressed the recently identified alphavirus receptor MxRA8 and granting viral entry and robust replication. A canonical innate immune response was engaged by ONNV-infected SC with elevated gene expression for RIG-I, MDA5, IFN-β, and ISG15 and inflammatory chemokine CCL5. Transcription levels of prostaglandin E2-metabolizing enzymes including cPLA2α, COX-2, and mPGES-1 were also upregulated in ONNV-infected SC. Counterintuitively, we found that ONNV failed to affect SC regenerative properties as indicated by elevated expression of the pro-myelinating genes MPZ and MBP1 as well as the major pro-myelin transcription factor Egr2. While ONNV infection led to decreased expression of CD55 and CD59, essential to control complement bystander cytotoxicity, it increased TRAIL expression, a major pro-apoptotic T cell signal. Anti-apoptotic Bcl2 transcription levels were also increased in infected SC. Hence, our study provides new insights regarding the remarkable immunomodulatory role of SC of potential importance in the pathogenesis of GBS following alphavirus infection.

## 1. Introduction

Alphaviruses are mosquito-borne enveloped viruses with a positive single-stranded RNA genome and they belong to the Togaviridae family. The alphavirus genomes encode four non-structural proteins (nsP1–nsP4), one capsid, two envelope glycoproteins (E1 and E2), and two peptides (E3 and 6K) using two open reading frames [1].

Canonically, alphaviruses are classified into two groups: the “old world” viruses, including Chikungunya virus (CHIKV), O’nyong’nyong (ONNV), and Sindbis and Ross River viruses, which cause predominantly rheumatic disorders; and the “new world” viruses, including Eastern, Western, and Venezuelan equine encephalitis viruses, which are mostly associated with neurological diseases [2]. CHIKV is closely related to ONNV, with which it shares 90% nucleotide sequence identity [3]. Although CHIKV is known to cause primarily arthritic disorders, CHIKV-associated neurologic complications have been recognized increasingly [4,5].

CHIKV-associated neuropathology was first reported in the 1960s [6]. However, the re-emergence of CHIKV infection in the 2000s in the Indian ocean regions with efficient clinical facilities allowed a better description of CHIKV-related neurological disease [7,8,9]. Whereas neurological complications mainly consisted of central nervous system (CNS)-associated disorders, Guillain–Barré syndrome (GBS) was the most common clinical presentation of peripheral nervous system (PNS) complications [10]. GBS is an acute inflammatory demyelinating disease of the PNS characterized by muscle weakness, autonomic dysfunctions, and sensory and motor perception impairment [11]. In most cases, GBS occurs after an infection and involves putative molecular mimicry mechanisms between pathogen and “self” antigens [12].

Schwann cells (SC) are the myelinating glial cells of the PNS. Myelination is a process by which SC produce a multi-layered membrane called the myelin sheath around the axonal membrane [12]. SC myelin cells express a large range of molecules that relate to the synthesis, maintenance, or structure of the myelin sheath. This includes the major pro-myelin transcription factor Egr2 and structural proteins such as myelin protein zero (MPZ) and myelin basic protein (MBP) [13]. In addition to their supportive role in PNS, SC also exert immune-like properties [14,15]. Indeed, they can recognize pathogen-associated molecular patterns (PAMPs) and host molecules containing damage-associated patterns (DAMPs), such as adenosine triphosphate (ATP) or S100, by expressing pattern recognition receptors (PRRs) such as TLR, RIG-I, and MDA5 [16,17]. Upon activation, SC initiate and regulate local immune responses by secreting cytokines and chemokines involved in immune cell recruitment to the site of injury [15,18]. Macrophage and T cell infiltration into the peripheral nerves has been reported to be associated with GBS [14,15,19].

Prostaglandins are a family of molecules involved in the regulation of inflammation [20]. They are synthesized from arachidonic acid, by the action of several enzymes including cyclooxygenase-2 (COX-2). Interestingly, increased COX-2 protein and prostaglandin E2 (PGE2) end product levels were detected in sera from patients with GBS, thereby supporting the implication of this inflammatory pathway in GBS pathogenesis [21].

The complement system is a major component of innate immunity. It is involved in the host defense against pathogens and the removal of immune complexes or apoptotic cells [22,23]. Complement activation via one of the three major pathways, the classical, lectin, and alternative pathways, leads to generation of the cytolytic membrane attack complex (MAC) [22,23]. A basal mechanism by which nucleated cells are protected from complement attack is the constitutive expression of cell surface membrane regulatory proteins, such as decay-accelerating factor (DAF; CD55) and CD59 [24]. 

Complement activation within the nervous system has been reported to contribute to the pathophysiology of GBS, as activation products of the complement cascade were found in serum and cerebrospinal fluid from patients with GBS [25,26].

We hypothesized that alphaviruses (including CHIKV and its close relative, ONNV) may be able to infect SC and modulate SC innate immune and inflammatory responses contributing to immune cell activation and recruitment, which may lead to peripheral nerve damage during CHIKV-associated GBS. We investigated human SC innate immune antiviral and inflammatory responses solely as a direct consequence of viral infection and compared this unique response to that of SC stimulated by canonical inflammatory molecules (cytokines) produced for instance by recruited immune cells. First, we used an in vitro model of primary SC culture infected with ONNV which served as a model of CHIKV infection and compared that to SC stimulated with the viral analog polyinosinic-polycytidylic acid (PIC). Moreover, to mimic the leukocyte-mediated inflammatory context, we stimulated SC with recombinant Interleukin-1β (Il-1β) or tumor necrosis factor-α (TNF-α). To investigate the role of DAMPs in peripheral neuropathology, we tested the direct effects of ATP on SC immune responses.

## 2. Materials and Methods

### 2.1. Cells, Virus, and Reagents

Human Schwann cells (SC) were obtained from ScienCell Research Laboratory (ScienCell, Carlsbad, CA, USA, 1700; Clinisciences). The primary cultures of SC were maintained in Minimum Essential Medium eagle (MEM Corning, NY, USA, 10-010-CV) supplemented with 10% decomplemented fetal bovine serum (FBS Gibco, E.U. Approved (South American Origin, Brazil), cat. no. 10270-106) and completed with L-glutamine 2 mM (PAN Biotech, Aidenbach, Germany, P04-08100), 100 U/mL–0.1 mg/mL penicillin-streptomycin (PAN Biotech, P0607100), 1 mM sodium pyruvate (PAN Biotech, P0443100), and 0.5 µg/mL fungizone (PAN Biotech, P0601001).

The double-stranded polyribonucleotide PIC (cat. no. 27-4732-01) was purchased from Amersham Biosciences (Amersham, UK). Interleukin-1β (IL-1β; cat. no. 200-01B), tumor necrosis factor-α (TNF-α; cat. no. 300-01A), and transforming growth factor-β2 (TGF-β2; cat. no. 100-35B) were purchased from Peprotech (London, UK). Adenosine triphosphate (ATP; cat. no. 9876557) was obtained from Biogems-Peprotech (Rehovot, Israel).

A clinical and passage-limited isolate of ONNV was obtained from the National Reference Center (CNR arbovirus, Marseille, France) and titrated on Vero cells at 10^7^ PFU/mL [27].

### 2.2. Cell Culture Treatment

SC were seeded in culture plates and incubated at 37 °C in a humid atmosphere with 5% CO_2_. When reaching 80–90% confluence, cells were infected with ONNV at different MOIs (1, 10^−1^, 10^−2^, and 10^−3^) or stimulated with the double-stranded PIC (10 µg/mL), ATP (1 mM), anti-inflammatory TGF-β2 (10 ng/mL), or the proinflammatory cytokines IL-1β (10 ng/mL) and TNF-α (10 ng/mL) for 6 h and 24 h. SC were exposed to extracellular PIC stimulation via addition to the culture medium. The concentrations used for the different stimuli were chosen from data published in the literature [28].

### 2.3. Cytotoxicity Assay

Cell damages were evaluated by measuring lactate dehydrogenase (LDH) release using a colorimetric-based kit (CytoTox 96 non-radioactive cytotoxicity assay, Promega, Madison, WI, USA). Supernatants of infected or treated cells were recovered and cells were lysed according to the manufacturer’s instructions. Cells lysed with 1% Triton X-100 were taken as the positive control and medium without cells as the negative control. Absorbance of converted dye was measured at 490 nm. Cytotoxicity was expressed relative to maximum LDH release with the formula: % cytotoxicity = 100 × experimental LDH release/maximum LDH release.

### 2.4. Quantitative Real-Time RT-PCR (qRT-PCR)

Total RNA was extracted from harvested cell culture using Zymo Research Quick-RNA Viral kit (Irvine, CA, USA, cat. no. R1035) according to the manufacturer’s instructions. qRT-PCR experiments were performed using the One Step Prime Script Syber Green RT-PCR kit from TAKARA (Cat. No RR066A). qRT-PCR was performed in a final volume of 5 μL containing 1 μL of extracted total RNA per reaction, 2.7 μL of enzyme mix, and 1.3 μL of primer mix with a final primer concentration of 250 nM. Primer pairs were designed in-house using the NCBI Primer Designing Tool (http://www.ncbi.nlm.nih.gov/tools/primer-blast/ accessed on 3 January 2020). The specific primers used are listed in Table 1.

qRT-PCR was carried out using a Quant Studio 5 thermocycler from Applied Biosystems (Thermo Fisher Scientific) with the following steps: a reverse transcription at 42 °C for 5 min followed by 40 cycles of a denaturation step at 95 °C for 5 s, an annealing step at 58 °C for 15 s, and an extension step at 72 °C for 15 s. Expression of the target mRNA was normalized to the expression of GAPDH mRNA.

### 2.5. Immunofluorescence Staining and Microscopy

SC were grown on glass coverslips and infected by ONNV at different MOIs for 24 h. Adherent cells were then fixed and permeabilized with frozen ethanol for 5 min and conserved at −20 °C. Coverslips were incubated with rabbit anti-CHIKV capsid (1:1000 dilution, a generous gift of Dr. A Merits, Estonia), mouse anti-MPZ (ABGent AT2897a, 1:500 dilution), Alexa Fluor 488-conjugated mouse anti-COX-2 (Santa Cruz sc-376861, 1:500 dilution), and Alexa Fluor 488-conjugated mouse anti-PGE synthase (PGES) (Santa Cruz sc-166308, 1:500 dilution). Alexa Fluor 488-conjugated donkey anti-mouse (Invitrogen A21202, 1:1000 dilution) and Alexa Fluor 594-conjugated donkey anti-rabbit (Invitrogen A21207, 1:1000 dilution) were used as secondary antibodies. Nuclei were revealed by staining with the nuclear fluorochrome 4′,6-diamidino-2-phenylindole (DAPI, Sigma-Aldrich, Darmstadt, Germany). The coverslips were mounted with Vectashield (Vector Labs; Clinisciences, Nanterre, France) and fluorescence was observed using a Nikon Eclipse E2000-U microscope (Nikon, Tokyo, Japan). Images were captured and processed using a Hamamatsu ORCA-ER camera and the imaging software NIS-Element AR (Nikon, Tokyo, Japan).

### 2.6. Enzyme-Linked Immune-Sorbent Assay (ELISA)

Chemokine concentrations in SC supernatants were measured using commercially available ELISA kits for CCL2 (Peprotech, London, UK: cat. no. 900-K31), CXCL8 (Peprotech; cat. no. 900-K18), and CCL5 (Peprotech; cat. no. 900-K33), according to the manufacturer’s instructions. Samples were analyzed from four independent experiments.

### 2.7. Statistics

Statistical analyses were performed with GraphPad Prism software version 6.01 using one-way ANOVA followed by the Bonferroni’s test for multiple comparisons. *p*-values ≤ 0.05 were considered statistically significant. Significance was indicated in the figures as follow: *p*-values ≤ 0.05 (*), *p*-values ≤ 0.01 (**), *p*-values ≤ 0.001 (***), and *p*-values ≤ 0.0001 (****). Results are expressed as mean ± standard error “SEM” and as percentages.

## 3. Results

### 3.1. The Alphavirus Receptor, MxRA8, Is Highly Expressed in ONNV-Infected SC

**Figure 1:**
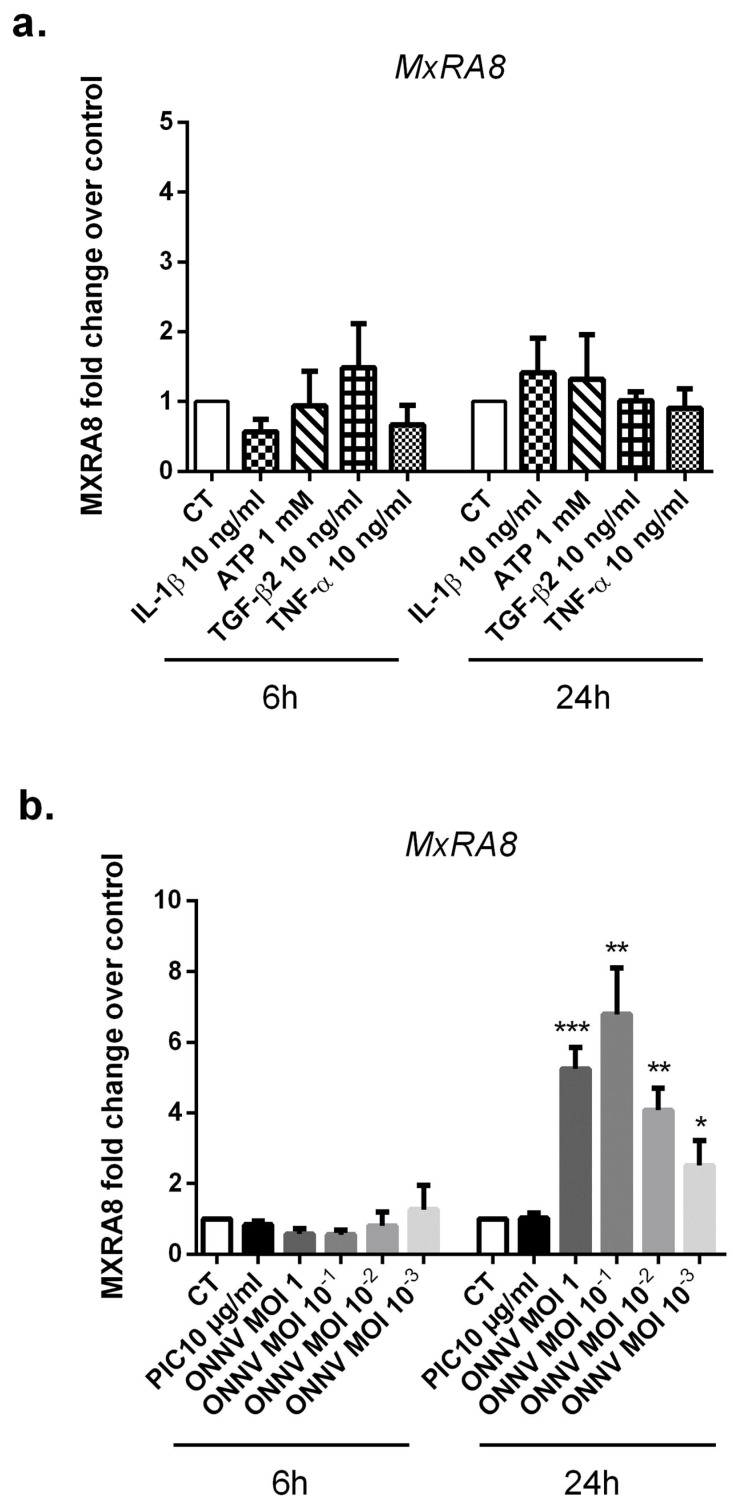
MxRA8 alphavirus entry receptor is upregulated in ONNV-infected SC. (**a**) MXRA8 mRNA levels from SC exposed to stimulatory treatments (IL-1β 10 ng/mL, TNF-α 10 ng/mL, TGF-β2 10 ng/mL, and ATP 1 mM) or (**b**) infected with ONNV (from MOI 10 ^−3^ to 1) or stimulated with PIC 10 µg/mL for 6 h and 24 h as assessed by qRT-PCR. All experiments were done in triplicates. Results are expressed as mean ± SEM and presented as a normalized fold increase vs. control (CT). * *p*-values ≤ 0.05, ** *p*-values ≤ 0.01, and *** *p*-values ≤ 0.001 represent significant differences from controls by one-way ANOVA followed by the Bonferroni’s test analysis.

Recently, the cell adhesion molecule MxRA8 has been identified as a receptor for multiple alphaviruses including CHIKV and ONNV [29]. We assessed by qRT-PCR the expression levels of MxRA8 in ONNV-infected SC as well as in response to different stimulatory conditions (Figure 1a,b). First, SC were infected with ONNV at a multiplicity of infection (MOI) 1, 10^−1^, 10^−2^, and 10^−3^ or stimulated with the viral analog PIC 10 µg/mL, ATP 1 mM, TGF-β2 10 ng/mL, or the proinflammatory cytokines IL-1β 10 ng/mL and TNF-α 10 ng/mL. Experiments were carried out for 6 h and 24 h. We found that cultured SC in control conditions expressed the alphavirus receptor MxRA8. The relative expression of MXRA8 in control cells was 2.03 × 10^−4^ ± 1.1 ×10^−4^ at 24 h.

ONNV infection strongly and significantly upregulated MxRA8 receptor mRNA levels at 24 h post infection, regardless of ONNV MOIs but with a five-fold increase with l ONNV MOI 1 (*p* < 0.01) compared to mock infected cells. We did not observe modulation of MxRA8 receptor expression under the following conditions: PIC, ATP, TGF-β2, IL-1β, and TNF-α.

### 3.2. SC Are Highly Susceptible to ONNV Infection

**Figure 2:**
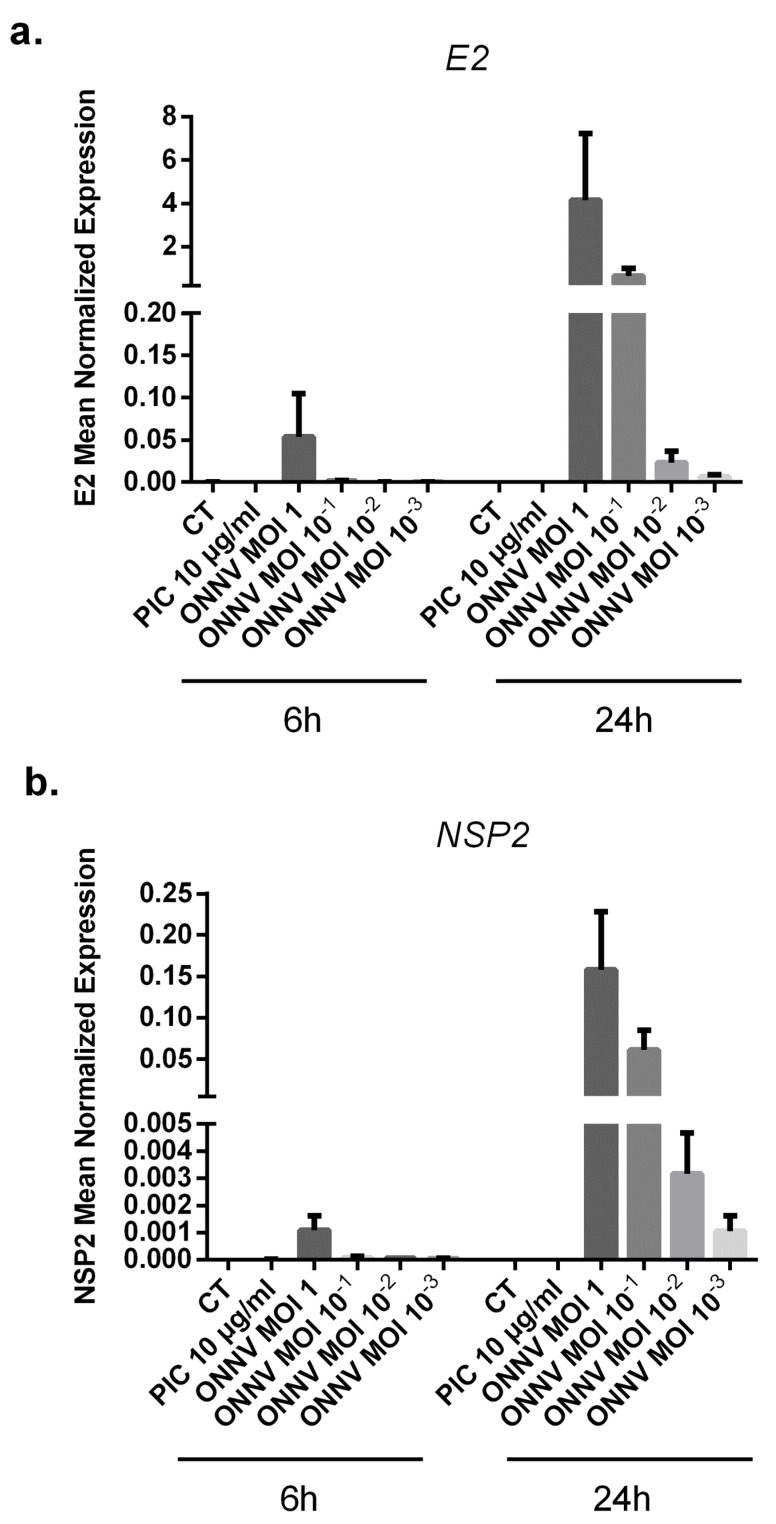
ONNV infects and replicates effectively in SC. (**a**) Relative expression of viral E2 and (**b**) NSP2 from infected SC with different MOIs of ONNV (from MOI 10^−3^ to 1) for 6 h and 24 h (qRT-PCR data). All experiments were done in triplicates.

To address whether SC are able to be infected and to replicate ONNV, SC cultures were challenged with increasing MOIs of ONNV (MOI 10^−3^, 10^−2^, 10^−1^, and 1) and the levels of two different viral RNA (E2 and NSP2) were quantified by qRT-PCR at 6 h and 24 h post infection (Figure 2a,b). Expression levels of E2 and NSP2 genes increased in infected SC at 24 h with the different tested MOIs. At 24 h post infection with ONNV MOI 1, the levels of viral E2 and NSP2 RNA increased approximately 100-fold more than at 6 h post infection (4.3 ± 5.17 × 10^−1^ and 1.58 × 10^−1^ ± 1.14 × 10^−1^, respectively, at 24 h compared to 5.2 × 10^−2^ ± 2.74 × 10^−2^ and 1.09 × 10^−3^ ± 7.47 × 10^−4^ at 6 h). Of note, we confirmed the infection and expression of high levels of capsid viral protein following CHIKV infection of SC (Figure S1).

### 3.3. ONNV Does Not Induce Cell Death in SC at Early Time Points

**Figure 3:**
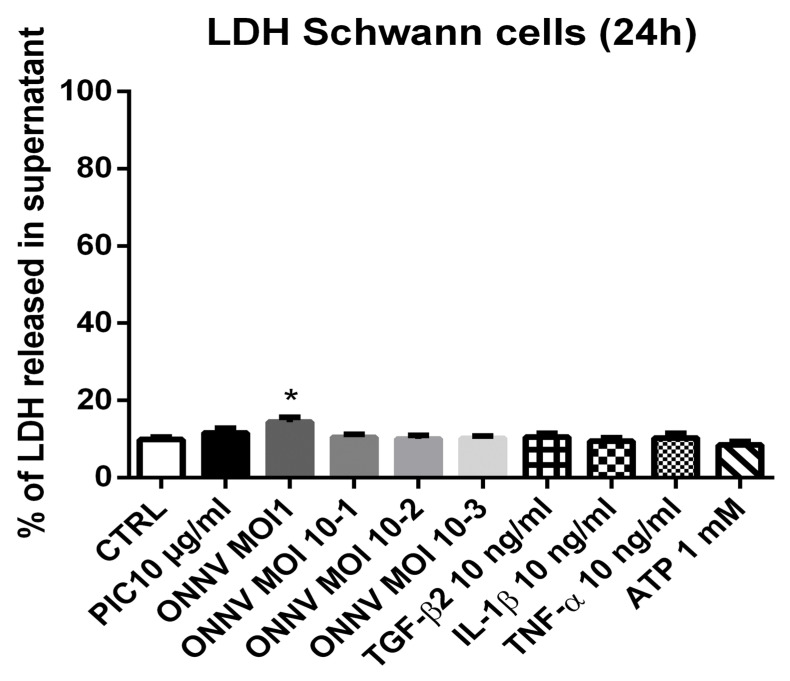
ONNV does not cause SC cytotoxicity at 24 h. SC were infected with ONNV (from MOI 10^−3^ to 1) or exposed to stimulatory treatments (PIC 10 µg/mL, IL-1β 10 ng/mL, TNF-α 10 ng/mL, TGF-β2 10 ng/mL, and ATP 1 mM) for 24 h. The percentage of LDH released in the culture supernatant was measured using the CytoTox 96 Non-Radioactive Cytotoxicity Assay. Results are from 4 independent experiments. * *p*-values ≤ 0.05 represent significant differences from controls (CT) by one-way ANOVA followed by the Bonferroni’s test analysis.

We assessed the capacity of ONNV and the different stimulatory treatments (PIC 10 µg/mL, IL-1β 10 ng/mL, TNF-α 10 ng/mL, TGF-β2 10 ng/mL, and ATP 1 mM) to impair SC cell viability by measuring the extracellular release of LDH. At 24 h post infection, ONNV MOI 1 slightly increased the level of LDH release in SC culture supernatants (14% ± 1.5% versus 10% ± 1% in mock infected cells, *p* < 0.05). ONNV at lower MOIs (10^−1^, 10^−2^, and 10^−3^), PIC 10 µg/mL, IL-1β 10 ng/mL, TNF-α 10 ng/mL, TGF-β2 10 ng/mL, and ATP 1 mM did not induce cellular cytotoxicity (Figure 3). However, prolonged infection with ONNV MOI 1, 10^−1^, and 10^−2^ caused significant cytotoxic effects on SC at 48 h post infection (67.3% ± 7.6%, *p* < 0.001; 55.9% ± 10.4%, *p* < 0.001 and 34.8% ± 7.9%, *p* < 0.01, respectively) (Figure S2).

### 3.4. SC Upregulate Antiviral Innate Immune-Related Genes in Response to ONNV Infection

**Figure 4:**
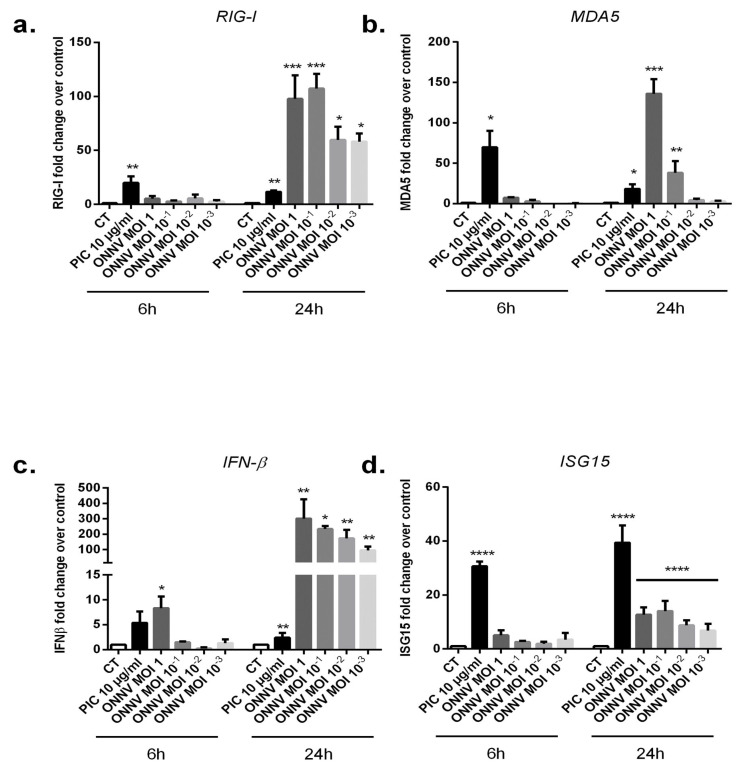
Canonical antiviral innate immune genes are markedly upregulated in ONNV-infected SC. (**a**) RIG-I, (**b**) MDA5, (**c**) IFN-β, and (**d**) ISG15 mRNA levels were quantified by qRT-PCR in SC after ONNV (from MOI 10^−3^ to 1) infection or exposure to PIC 10 µg/mL for 6 h and 24 h. All experiments were done in triplicates. Results are expressed as mean ± SEM and presented as a normalized fold increase vs. control (CT). * *p*-values ≤ 0.05, ** *p*-values ≤ 0.01, *** *p*-values ≤ 0.001, and **** *p*-values ≤ 0.0001 represent significant differences from controls by one-way ANOVA followed by the Bonferroni’s test analysis.

We evaluated the capacity of ONNV to modulate the expression of several genes encoding for proteins having a key antiviral role. We thus carried out qRT-PCR to screen for the expression of RLRs (RIG-I and MDA5) as well as IFN-β and ISG15 in SC at 6 h and 24 h post infection. First, in response to PIC stimulation (Figure 4a,b), the relative mRNA expression of the virus sensors RIG-I and MDA5 were significantly increased in SC at 6 h and 24 h. At 6 h post PIC treatments, the levels of expression were (3.5 × 10^−2^ ± 1.48 × 10^−2^, *p* < 0.01) for RIG-I with a fold increase of 20 and (2.26 × 10^−3^ ± 2.23 × 10^−3^, *p* < 0.05) for MDA5 with a fold increase of 80 compared to control conditions (1.75 × 10^−3^ ± 5.44 × 10^−4^ for RIG-I and 2.71 × 10^−5^ ± 1.67 × 10^−5^ for MDA5). The mRNA levels of IFN-β and ISG15 were also significantly upregulated after PIC stimulation (6.88 × 10^−5^ ± 1.61 × 10^−5^, *p* < 0.01) for IFN-β (2.5-fold) and (3.84 ± 1.43, *p* < 0.0001) for ISG15 (38-fold) at 24 h compared to control conditions (2.48 × 10^−5^ ± 1.36 × 10^−5^ for IFN-β and 1 × 10^−1^ ± 3.45 × 10^−2^ for ISG15) (Figure 4c,d).

Interestingly, ONNV at the different tested MOIs strongly increased the expression of the antiviral innate immune genes RIG-I, MDA5, IFN-β, and ISG15 with a significant and more robust effect at 24 h post infection. For instance, ONNV MOI 10^−1^ upregulated RIG-I mRNA levels in SC up to a 104-fold increase (8.41 × 10^−2^ ± 3.05 × 10^−2^, *p* < 0.001), MDA5 expression levels up to a 38-fold increase (6.99 × 10^−3^ ± 4.86 × 10^−3^, *p* < 0.01), IFN-β up to a 234-fold increase (5.8 × 10^−3^ ± 1.17 × 10^−3^, *p* < 0.05), and ISG15 up to 16-fold increase (1.61 ± 1.01, *p* < 0.0001) at 24 h post infection. The levels of expression in mock infected cells at 24 h were: 8.06 × 10^−4^ ± 3.29 × 10^−4^ for RIG-I; and 1.88 × 10^−4^ ± 1.11 × 10^−4^ for MDA5.

### 3.5. SC Induce a Robust Chemokine Response Following ONNV Infection and after IL-1β or TNF-α Stimulation

Chemokines are important mediators of inflammation known to be involved in chemotaxis and the activation of phagocytes and lymphocytes [30]. Inflammatory chemokines and infiltration of activated monocytes and lymphocytes are described as key events in the pathogenesis of GBS [17,31]. In particular, CCL2, CXCL8, and CCL5 chemokines are known to be principally involved in the recruitment of macrophages, neutrophils, and T cells, respectively.

**Figure 5:**
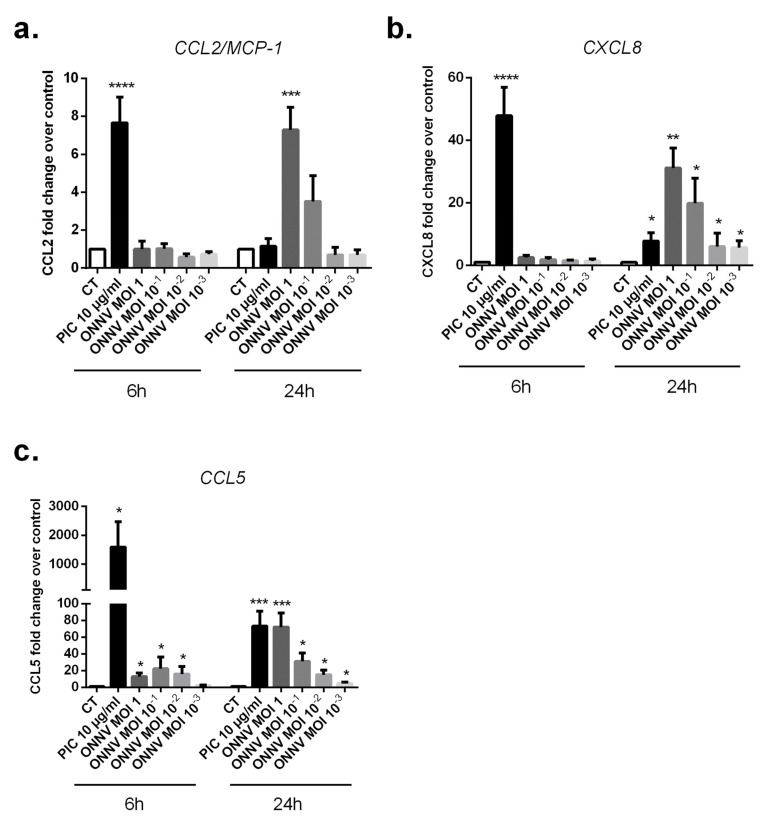
Marked upregulation of proinflammatory chemokines by SC in response to ONNV infection and PIC stimulation. (**a**) CCL2, (**b**) CXCL8, and (**c**) CCL5 mRNA levels from SC after ONNV (MOI 10^−3^ to 1) infection or exposure to PIC 10 µg/mL for 6 h and 24 h, as measured by qRT-PCR. All experiments were done in quadruplicates. Results are expressed as mean ± SEM and presented as a normalized fold increase vs. control (CT). * *p*-values ≤ 0.05, ** *p*-values ≤ 0.01, *** *p*-values ≤ 0.001, and **** *p*-values ≤ 0.0001 represent significant differences from controls by one-way ANOVA followed by the Bonferroni’s test analysis.

We therefore evaluated the capacity of ONNV, PIC, and the other stimulatory treatments (IL-1β 10 ng/mL, TNF-α 10 ng/mL, TGF-β2 10 ng/mL, and ATP 1 mM) to induce the expression of CCL2, CXCL8, and CCL5 by SC. We have first investigated by qRT-PCR, the effects of PIC and ONNV on CCL2, CXCL8, and CCL5 mRNA levels in SC (Figure 5a–c). PIC significantly upregulated mRNA expression of all tested chemokines at 6 h and 24 h. More than a seven-fold increase was observed at 6 h for CCL2 (2.65 × 10^−1^ ± 1.14 × 10^−1^, *p* < 0.0001) versus (3.3 × 10^−2^ ± 1.02 × 10^−2^), a 48-fold increase for CXCL8 (1.83 × 10^−1^ ± 8.07 × 10^−2^, *p* < 0.0001) versus (3.82 × 10^−3^ ± 1.56 × 10^−3^), and more than a 1400-fold increase was detected for CCL5 (5.98 × 10^−2^ ± 4.04 × 10^−2^, *p* < 0.05) versus (4.18 × 10^−5^ ± 1.58 × 10^−5^).

The capacity of ONNV to modulate CCL2, CXCL8, and CCL5 expressions was next observed essentially at 24 h post infection with a seven-fold increase for CCL2 mRNA levels (3.34 × 10^−1^ ± 2.43 × 10^−1^, *p* < 0.001) versus (4.67 × 10^−2^ ± 3.30 × 10^−2^), a 33-fold increase for CXCL8 mRNA levels (2.26 × 10^−2^ ± 1.33 × 10^−2^, *p* < 0.01) versus (6.76 × 10^−4^ ± 4.90 × 10^−4^), and a 72-fold increase for CCL5 mRNA levels (5.06 × 10^−3^± 2.04 × 10^−3^, *p* < 0.001) versus (7.02 × 10^−5^ ± 3.3 × 10^−5^) in response to ONNV MOI 1 infection.

**Figure 6:**
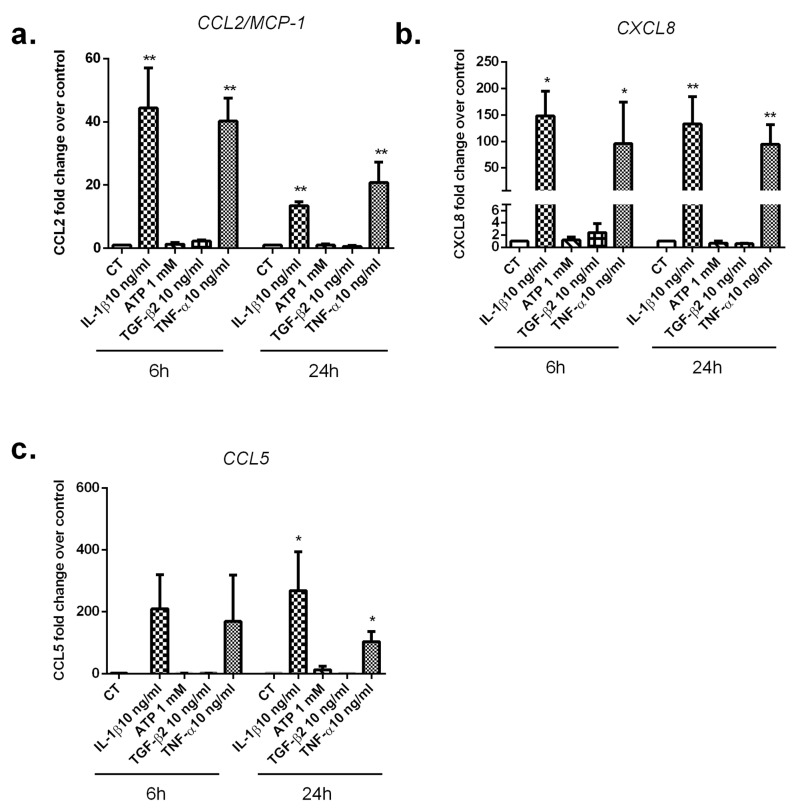
Regulated mRNA expression of proinflammatory chemokines in response to IL-1β, TNF-α, TGF-β2, and ATP stimulations in SC. (**a**) Relative expression of CCL2, (**b**) CXCL8, and (**c**) CCL5 from SC stimulated by IL-1β, TNF-α, TGF-β2, and ATP for 6 h and 24 h, as assessed by qRT-PCR. All experiments were done in triplicates. Results are expressed as mean ± SEM and presented as a normalized fold increase vs. control (CT). * *p*-values ≤ 0.05 and ** *p*-values ≤ 0.01 represent significant differences from controls by one-way ANOVA followed by the Bonferroni’s test analysis.

We next evaluated the stimulatory effects of the pro-inflammatory cytokines IL-1β and TNF-α and the anti-inflammatory growth factor TGF-β2 and ATP (DAMP) on CCL2, CXCL8, and CCL5 expressions (Figure 6a–c).

When exposed to IL-1β and TNF-α, SC significantly increased the expression of all tested chemokines at 24 h. CCL2 mRNA levels were increased by about 13-fold post-IL-1β stimulation (*p* < 0.01) and 21-fold post-TNF-α stimulation (*p* < 0.01). Up to a 133-fold increase was detected on CXCL8 mRNA levels after IL-1β stimulation (*p* < 0.01) and up to a 95-fold increase in response to TNF-α stimulation (*p* < 0.01). CCL5 mRNA levels were increased by about 260-fold post-IL-1β stimulation (*p* < 0.05) and 100-fold post-TNF-α stimulation (*p* < 0.05). However, TGF-β2 and ATP failed to modulate CCL2, CXCL8, and CCL5 expressions in SC at 6 h and 24 h.

**Figure 7:**
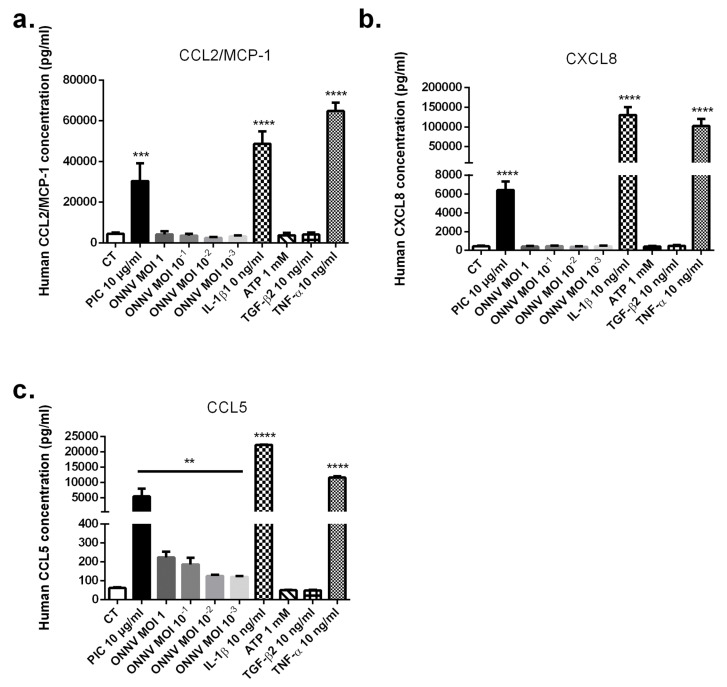
SC can modulate proinflammatory chemokine production in response to PIC and proinflammatory cytokines (IL-1β and TNF-α) stimulation and ONNV infection. SC were infected with ONNV (from MOI 10^−3^ to 1) or exposed to stimulatory treatments (PIC 10 µg/mL, IL-1β 10 ng/mL, TNF-α 10 ng/mL, TGF-β2 10 ng/mL, and ATP 1 mM). Supernatants were harvested after 24 h and levels of CCL2 (**a**), CXCL8 (**b**), and CCL5 (**c**) were quantitated by ELISA assay. All experiments were done in quadruplicates and results are expressed as mean ± standard error. ** *p*-values ≤ 0.01, *** *p*-values ≤ 0.001, and **** *p*-values ≤ 0.0001represent significant differences from controls by one-way ANOVA followed by the Bonferroni’s test analysis.

Chemokine protein levels were then monitored in SC culture supernatants. The upregulation of CCL2, CXCL8, and CCL5 expressions after PIC, IL-1β, and TNF-α exposure was confirmed at the protein level by ELISA assay at 24 h (Figure 7a–c). For instance, PIC stimulation significantly increased CCL2 concentration (30,442 pg/mL ± 5015, *p* < 0.001) versus (4495 pg/mL ± 617) with a fold change of 7, CXCL8 concentration (6414 pg/mL ± 1553, *p* < 0.0001) versus (443 pg/mL ± 101) with a fold change of 14, and CCL5 concentration (5413 pg/mL ± 1903, *p* < 0.01) versus (61 pg/mL ± 5.8) with a fold change of 89 at 24 h. Among the tested chemokines, only CCL5 protein levels were upregulated in response to ONNV (from MOI 10^−3^ to 1) infection at 24 h with a fold increase of 4 (223 pg/mL ± 50.8, *p* < 0.01) after ONNV MOI 1 infection. ONNV failed to increase CCL2 and CXCL8 protein levels at 24 h and also at 30 h post infection (Figure S3).

### 3.6. Regulated Expression of Prostaglandin E2-Metabolic Pathway in Cytokine-Stimulated and ONNV-Infected SC

We assessed the capacity of SC to modulate the expression of several enzymes of the PGE2 biosynthetic pathway, including cPLA2α, mPGES-1, COX-2, and 15-PGDH. Cellular responses were evaluated after ONNV infection and PIC, IL-1β, TNF-α, TGF-β2, and ATP exposure.

**Figure 8:**
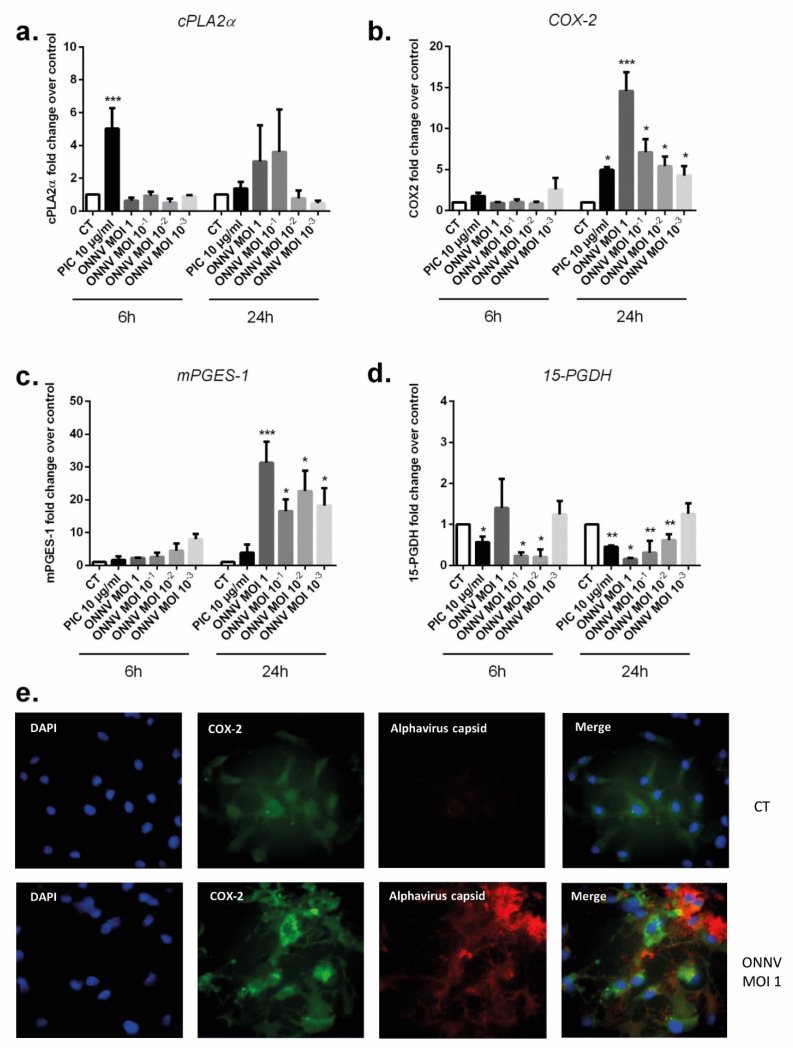
SC can modulate prostaglandin E2-biosynthesis pathway after ONNV infection and PIC stimulation. (**a**) cPLA2α, (**b**) COX-2, (**c**) mPGES-1, and (**d**)15-PGDH mRNA levels were evaluated by qRT-PCR in SC infected by ONNV (MOI 10-3 to 1) or exposed to PIC 10 µg/mL for 6 h and 24 h. All experiments were done in quadruplicates. Results are expressed as mean ± SEM and presented as a normalized fold increase vs. control. * *p*-values ≤ 0.05, ** *p*-values ≤ 0.01, and *** *p*-values ≤ 0.001 represent significant differences from controls by one-way ANOVA followed by the Bonferroni’s test analysis. (**e**) SC were cultured on coverslips, incubated with ONNV (MOI 1) for 24 h, or mock infected (CT) and analyzed by fluorescence microscopy. Immunostaining was performed using rabbit anti-CHIKV capsid and Alexa Fluor 594 (red)-conjugated donkey anti-rabbit or Alexa Fluor 488 (green)-conjugated mouse anti-COX-2. Nuclei were counterstained with DAPI (blue).

PIC significantly enhanced mRNA expression of the PGE2 synthesizing enzymes cPLA2α (five-fold increase at 6 h, *p* < 0.001) and COX-2 (five-fold increase at 24 h, *p* < 0.05) and decreased mRNA expression of the PGE2 degrading enzyme 15-PGDH (two-fold decrease at 24 h, *p* < 0.01) (Figure 8a,b,d).

Transcription levels of the PGE2-synthesizing enzymes mPGES-1 and COX-2 were also upregulated in ONNV-infected SC at the different tested MOIs (from 10^−3^ to 1) at 24 h post infection. When infected with ONNV MOI 1, SC upregulated mPGES-1 mRNA levels up to 31-fold (6.27 × 10^−4^ ± 4.11 × 10^−4^, *p* < 0.001) versus (2.03 × 10^−5^ ± 1.69 × 10^−5^) and COX-2 mRNA levels up to 15-fold (2.36 × 10^−2^ ± 2.29 × 10^−2^, *p* < 0.001) versus (1.48 × 10^−3^ ± 1.38 × 10^−3^) at 24 h (Figure 8b,c). In contrast, 15-PGDH gene expression was significantly decreased by about five-fold (8.2 × 10^−5^ ± 3.75 × 10^−5^, *p* < 0.05) versus (3.9 × 10^−4^ ± 1.69 × 10^−4^) in response to ONNV MOI 1 infection at 24 h (Figure 8d).

To support qRT-PCR data, we performed immunofluorescence to detect and evaluate the expression level of COX-2 in ONNV-infected SC (Figure 8e). ONNV-infected cells visually led to enhanced COX-2 detection in neighboring cells. ONNV infection also increased mPGES-1 detection in SC compared to mock infected cells (Figure S4).

We also tested the capacity of the proinflammatory cytokines IL-1β and TNF-α to upregulate PGE2 synthesizing enzymes. SC stimulation by IL-1β 10 ng/mL and TNF-α 10 ng/mL markedly enhanced cPLA2α, mPGES-1, and COX-2 mRNA levels. For instance, IL-1β 10 ng/mL increased cPLA2α mRNA levels by about three-fold (*p* < 0.01), mPGES-1 mRNA expression by about nine-fold (*p* < 0.01), and COX-2 mRNA levels by about seven-fold (*p* < 0.001) at 6 h. IL-1β 10 ng/mL also decreased 15-PGDH mRNA levels by about three-fold at 24 h (*p* < 0.0001) (Figure S5).

### 3.7. SC Modulate the Expression of Myelin-Related Genes in Response to ONNV Infection

**Figure 9:**
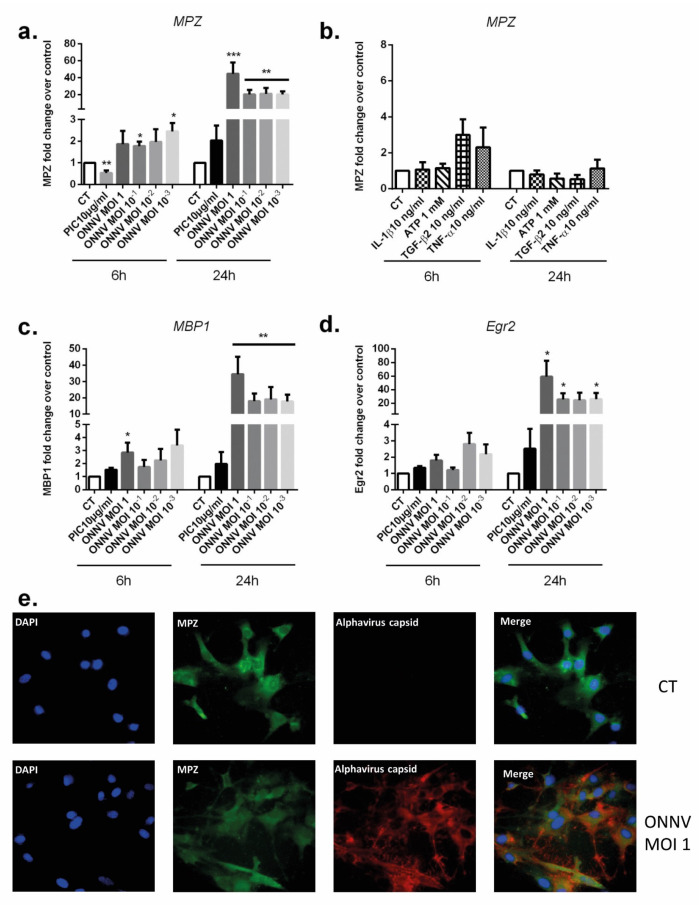
SC upregulated the expression of myelin-related genes in response to ONNV infection. (**a**) MPZ mRNA levels were assessed by qRT-PCR in SC infected by ONNV (MOI 10^−3^ to 1) or exposed to PIC 10 µg/mL, (**b**) IL-1β 10 ng/mL, TNF-α 10 ng/mL, TGF-β2 10 ng/mL, and ATP 1 mM. (**c**) MBP1 and (**d**) Erg2 gene expression were quantified by qRT-PCR in SC infected by ONNV or stimulated by PIC 10 µg/mL for 6 h and 24 h. All experiments were done in quadruplicates. Results are expressed as mean ± SEM and presented as a normalized fold increase vs. control. * *p*-values ≤ 0.05, ** *p*-values ≤ 0.01, and *** *p*-values ≤ 0.001 represent significant differences from controls by one-way ANOVA followed by the Bonferroni’s test analysis. (**e**) SC were cultured on coverslips, incubated with ONNV (MOI 1) for 24 h, or mock infected (CT) and analyzed by fluorescence microscopy. Immunostaining was performed using rabbit anti-CHIKV capsid and Alexa Fluor 594 (red)-conjugated donkey anti-rabbit or mouse anti-MPZ and Alexa Fluor 488 (green)-conjugated donkey anti-mouse. Nuclei were counterstained with DAPI (blue).

The myelin sheath produced by SC is essential for efficient nerve transmission in the PNS. As previously described, GBS is characterized by a progressive loss of myelin sheath which thus disrupts nerve transmission in peripheral nerves [11]. SC are required for successful nerve regeneration during peripheral nerve injuries [32]. SC differentiation into mature myelinating cells is pivotal for remyelination. We wondered if ONNV infection may alter SC regenerative properties and prevent their differentiation into myelinating cells by downregulating pro-myelinating genes. Therefore, we evaluated the expression of the pro-myelinating genes MPZ, MBP1, and the major pro-myelin transcription factor Egr2 in SC in response to ONNV infection or PIC exposure (Figure 9a,c,d). Interestingly, ONNV infection strongly increased mRNA levels of MPZ by about 45-fold (8.15 × 10^−1^ ± 8.88 × 10^−2^, *p* < 0.001), MBP1 by about 34-fold (2.91 × 10^−2^ ± 1.70 × 10^−2^, *p* < 0.01), and Egr2 by 59-fold (3.71 × 10^−1^ ± 1.85 × 10^−1^, *p* < 0.05) at 24 h post infection compared to mock infected cells (1.79 × 10^−2^ ± 1.08 × 10^−2^ for MPZ, 8.57 × 10^−4^ ± 5.83 × 10^−4^ for MBP1, and 6.28 × 10^−3^ ± 3.74 × 10^−3^ for Egr2). In contrast, PIC treatment notably decreased MPZ gene expression at 6 h post infection compared to untreated cells (two-fold decrease, *p* < 0.01). Proinflammatory cytokines IL-1β and TNF-α, TGF-β2, and ATP exposure had no significant effect on pro-myelinating gene expression in SC (Figure 9b).

To support qRT-PCR data, we performed immunofluorescence to detect MPZ expression in ONNV-infected SC (Figure 9e). By immunofluorescence assay, we showed a slightly more intense fluorescence for the structural protein MPZ in ONNV-infected SC compared to mock infected cells. Collectively and counterintuitively, we can conclude that ONNV infection upregulated pro-myelinating MPZ, MBP1, and Egr2 expression in SC.

### 3.8. Regulated Expression of Complement Regulatory Proteins CD55 and CD59 in ONNV-Infected SC

The complement system is an essential component of the innate immune response. It is involved in pathogen recognition and immunoregulation [22,23]. Aberrant complement cascade activation on “self” membranes can be prevented by several regulators such as: decay-accelerating factor (CD55), which inhibits C3 and C5 convertase formation, and CD59, which prevents terminal cytolytic membrane attack complex formation. Some viruses were reported to avoid complement destruction by modulating the expression of complement regulatory proteins [33]. 

**Figure 10:**
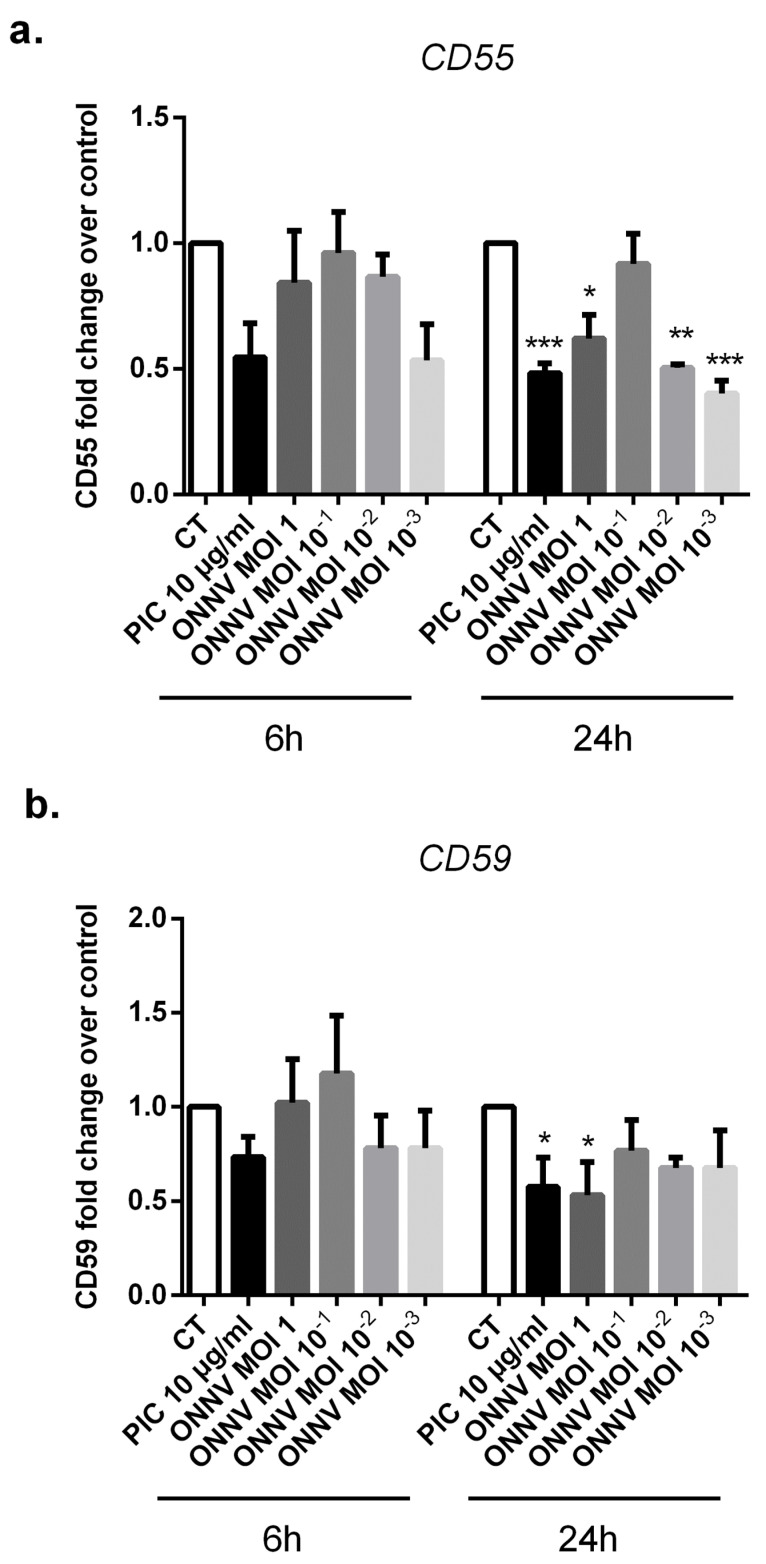
mRNA expression of two major complement regulatory proteins CD55 and CD59 are downregulated after ONNV infection. (**a**) CD55 and (**b**) CD59 mRNA levels were quantified by qRT-PCR in SC following ONNV (MOI 10^−3^ to 1) infection or exposure to PIC 10 µg/mL for 6 h and 24 h. All experiments were done in triplicates. Results are expressed as mean ± SEM and presented as a normalized fold increase vs control. * *p*-values ≤ 0.05, ** *p*-values ≤ 0.01, and *** *p*-values ≤ 0.001 represent significant differences from controls by one-way ANOVA followed by the Bonferroni’s test analysis.

We evaluated the capacity of ONNV (from MOI 10^−3^ to 1) and the viral analog PIC to regulate the expression of the complement regulatory proteins CD55 and CD59 in SC (Figure 10a,b). PIC significantly downregulated CD55 expression levels (1.91 × 10^−3^ ± 1.47 × 10^−3^, *p* < 0.001) (three-fold decrease) and CD59 expression levels (1.84 × 10^−2^ ± 5.93 × 10^−3^, *p* < 0.05) (two-fold decrease) at 24 h post-stimulation compared to control cells (5.73 × 10^−3^ ± 3.69 × 10^−3^) and (3.35 × 10^−2^ ± 1.2 × 10^−2^), respectively. In response to ONNV MOI 1, SC reduced CD55 and CD59 gene expression by about two-fold (2.89 × 10^−3^ ± 1.41 × 10^−3^, *p* < 0.05) and three-fold (1.19 × 10^−2^ ± 3.31 × 10^−3^, *p* < 0.05), respectively, at 24 h post infection. CD55 mRNA levels were also downregulated in the presence of lower ONNV MOIs (MOI 10^−2^ and 10^−3^). 

### 3.9. SC Modulate the Expression of the Proapoptotic Ligand TRAIL and the Apoptotic Regulator Bcl 2 in Response to ONNV Infection

**Figure 11:**
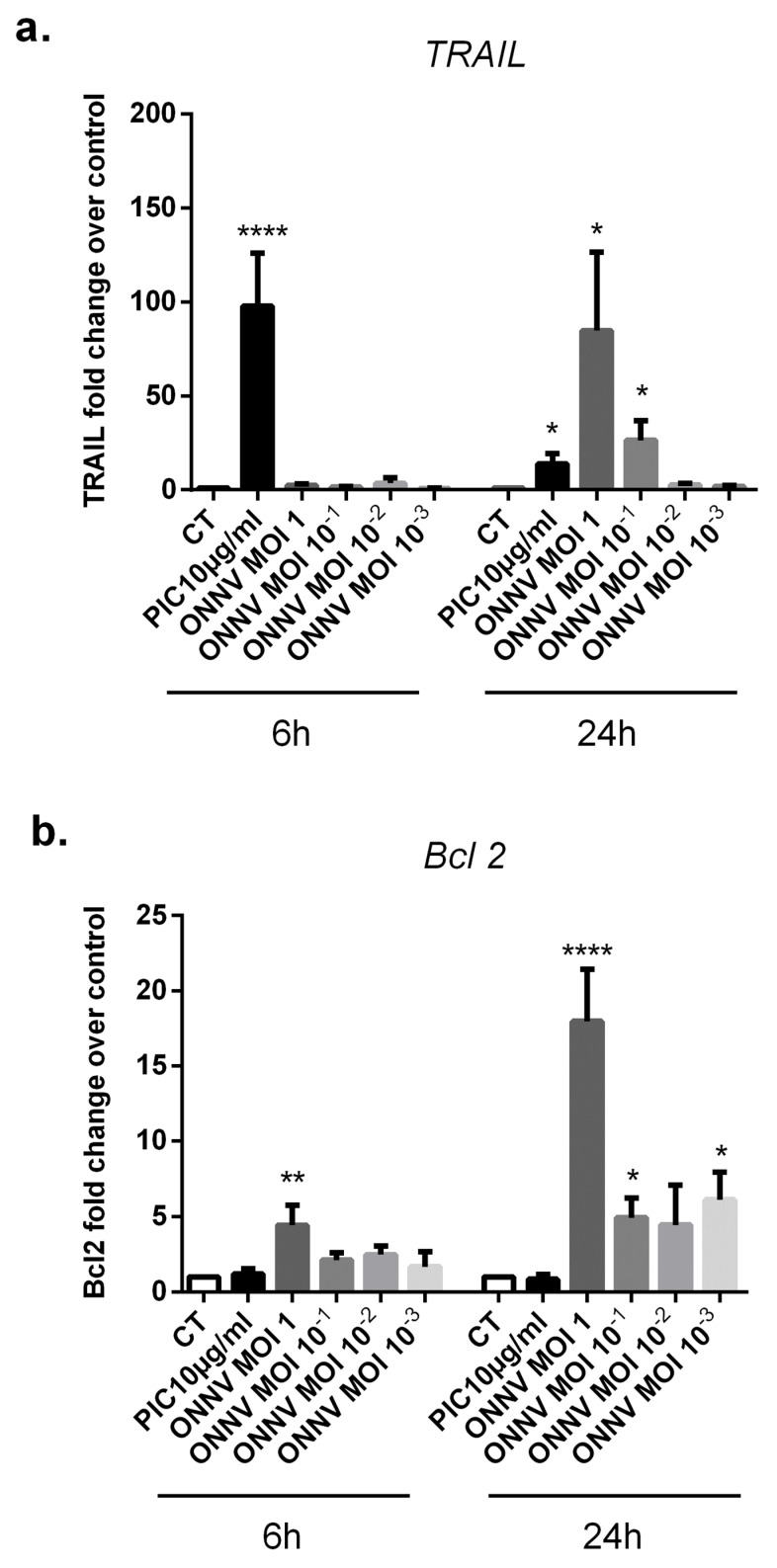
SC upregulate TRAIL and Bcl 2 mRNA expression in response to ONNV infection. (**a**) TRAIL and (**b**) Bcl 2 mRNA levels were assessed by qRT-PCR in SC infected by ONNV (MOI 10^−3^ to 1) or exposed to PIC 10 µg/mL for 6 h and 24 h. All experiments were done in triplicates. Results are expressed as mean ± SEM and presented as a normalized fold increase vs. control. * *p*-values ≤ 0.05, ** *p*-values ≤ 0.01, and **** *p*-values ≤ 0.0001 represent significant differences from controls by one-way ANOVA followed by the Bonferroni’s test analysis.

Apoptosis is a tightly regulated form of controlled cellular self-destruction. The induction of apoptosis or programmed cell death in virus-infected cells is an important antiviral defense mechanism [34]. We assessed the expression of the proapoptotic factor TNF-related, apoptosis-inducing ligand (TRAIL) and the anti-apoptotic protein B-cell lymphoma 2 (Bcl 2) in ONNV-infected and PIC-stimulated SC (Figure 11a,b). Our results showed that PIC strongly upregulated TRAIL mRNA levels up to 75-fold at 6 h (1.77 × 10^−2^ ± 1.17 × 10^−2^, *p* < 0.0001) compared to control cells (2.36 × 10^−4^ ± 1.22 × 10^−4^). However, Bcl 2 gene expression was not modulated by PIC at 6 h and 24 h.

Interestingly, ONNV strongly upregulated TRAIL mRNA levels but also Bcl 2 gene expression. TRAIL mRNA levels were increased up to 85-fold (4.19 × 10^−2^ ± 1.32 × 10^−2^, *p* < 0.05) compared to mock infected cells (4.94 × 10^−4^ ± 2.15 × 10^−4^), whereas Bcl 2 mRNA levels increased up to 18-fold (1.07 × 10^−2^ ± 1.03 × 10^−2^, *p* < 0.0001) versus (6 × 10^−4^ ± 2.93 × 10^−4^) in mock infected cells at 24 h post ONNV MOI 1 infection. 

## 4. Discussion

Alphavirus infection has been reported to trigger the development of GBS in some patients through an ill-characterized mechanism. Acute and rapidly progressive flaccid peripheral paralysis may be driven through a localized inflammatory response orchestrated by recruited immune cells and/or compromised SC [35]. However, the direct or indirect mechanisms involved in peripheral neuropathology remain largely unknown. Indeed, alphavirus may induce GBS by damaging SC either directly or through the robust innate and adaptive immune inflammatory responses against the virus. We used the alphavirus ONNV to better investigate SC innate immune antiviral and inflammatory responses potentially protecting the nerve or contributing further to the injury. We demonstrated for the first time that SC express the recently discovered alphavirus receptor, MxRA8 [29], whose expression was significantly increased in response to ONNV infection. It has been shown that the deletion of MxRA8 gene or blocking of the surface protein in human and murine cells reduced alphaviral infection. MxRA8 was found to bind directly to CHIKV E2 protein and enhance virus attachment and internalization into the cells [29,36]. MxRA8, initially known as limitrin (also called DICAM or ASP3), is an adhesion molecule expressed on epithelial, myeloid, and mesenchymal cells [37,38,39]. The role of MxRA8 in the peripheral nerve is yet to be identified. In the CNS, MxRA8 was found to be highly expressed in astrocyte endfeet (glia limitans) where it was suggested to regulate the maturation and efficacy of the blood brain barrier [39]. Of note, astrocytes are highly susceptible to CHIKV infection and link to nervous tissue invasion [40]. Whether MxRA8 has a role at the level of the blood peripheral nerve barrier is unknown. Further studies using specific MXRA8 antagonists will need to address the role of the alphavirus receptor MxRA8 in downstream signaling events in Schwann cells. Studying these different post infectious signaling cascades also opens the way to new therapies, targeting in particular MxRA8 receptor of intracellular pathways.

We showed that primary SC are susceptible to ONNV infection associated with elevated expression of E2 and NSP2 viral genes between 6 h and 24 h post infection. CHIKV was reported to be able to infect a wide variety of neuroglial cell types including neurons, astrocytes, and oligodendrocytes in the CNS, but the infection of SC remained to be tested [40,41]. Murine in vivo infection studies showed that CHIKV can efficiently replicate in the brain of newborn mice, reaching high viral titers of 10^6^–10^7^ PFU/mL [40,42]. Here, we demonstrated for the first time the ability of alphaviruses (ONNV but also CHIKV, data not shown) to infect the PNS-related glia cells, the SC.

Following ONNV infection, SC were able to initiate a robust antiviral response by upregulating the mRNA expression of the two major pattern recognition receptors RIG-I and MDA5 but also mRNA levels of antiviral IFN-β and the downstream IFN-stimulated antiviral gene ISG15. SC are known as immune competent cells and can express a broad range of pattern recognition receptors that allows them to recognize exogenous as well as endogenous danger signals [18]. In a previous in vitro study, SC were found to be able to initiate a robust antiviral response to infection with flaviviruses Zika virus (ZIKV) and yellow fever virus (YFV) [43].

In addition to their ability to activate an antiviral response, we showed that SC upregulated chemokines production including CXCL8, CCL5, and CCL2 under proinflammatory conditions. Cytokines and chemokines have been described to play a pivotal role in the pathogenesis of GBS. Chemokines are involved in chemotaxis and activation of phagocytes and lymphocytes. Macrophage and T cell infiltration into peripheral nerves and spinal nerve roots has been associated with GBS [19]. Several chemokines, such as CCL2 and CCL5, were found to be upregulated in experimental model of GBS [44]. CCL2 chemokine allows the recruitment of macrophages that can actively participate to the destruction of myelin [45]. Using an in vivo model of GBS, Orlikowski and colleagues observed a significant infiltration of macrophages in peripheral nerves of affected mice. Similarly, they detected a strong macrophages infiltration in nerve biopsies of GBS patients with elevated CCL2 concentrations in the serum of patients [30]. Elevated levels of both CCL2 and CCL5 were also detected in a mice experimental model of GBS at expected peak clinical severity [46]. Using immunohistochemical techniques, Xia et al. demonstrated that CCL2 primarily colocalized to SC and CCL5 to axons and suggested that CCL2 may be required for macrophage-dependent demyelination or SC injury, whereas CCL5 may be required for axonal degeneration [46]. We showed that ONNV infection increased CCL5 but not CCL2 and CXCL8 production, suggesting that ONNV-induced inflammatory response in SC can potentially lead to a T-dependent rather than a macrophage- or neutrophil-dependent response.

Interestingly, we also demonstrated that ONNV-infected SC showed significant activation of the prostaglandin pathway by upregulating the expression of cPLA2α, mPGES-1, and COX-2 mRNA. PGE2 is known as an important lipid mediator that is tied closely with inflammatory processes [20]. COX-2, a key enzyme of the prostaglandin biosynthetic pathway, was found to be highly expressed in SC and activated macrophages in a nerve injury in vivo model [47]. Interestingly, increased COX-2 protein levels were detected in serum and cerebrospinal fluid of GBS patients. Furthermore, increased levels of PGE2 were measured in sera from patients with GBS [21]. Targeting inflammatory pathways with anti-inflammatory drugs would therefore be beneficial for both the acute (GBS) and chronic (arthritis) phases of alphavirus infections [48].

Another essential component of the innate immune response is the complement system. The complement system is involved in the host defense against pathogens and the removal of immune complexes or apoptotic cells [22]. The complement system has been demonstrated to have both an antiviral and pathogenic role during alphavirus infection. For instance, the complement system has been shown to limit viremia and reduce the brain amount of Sindbis virus in infected mice while shortening mouse survival time after infection [49]. Some viruses were reported to avoid complement destruction by modulating the expression of complement regulatory proteins [33]. Hepatitis C virus was shown to upregulate the expression of CD55 onto the hepatocyte [50]. Complement regulatory proteins CD55 and CD59 were found to be expressed in SC and oligodendrocytes [51]. CD59 was detected on SC membranes, satellite cells in spinal ganglia, and endothelial cells in GBS and control patients’ tissues [52]. CD59 expression was found to be upregulated at sites of myelin disruption on the human material, supporting a protective role of CD59 in inflammatory diseases of the PNS [52]. We showed that ONNV reduced CD55 and CD59 expression in infected SC. Decreased CD55 and CD59 expression in ONNV-infected SC may potentially contribute to bystander complement activation, myelin opsonization C3b opsonins, and phagocytosis by macrophages through complement surface receptor CR3 and possibly contribute to SC complement-mediated lysis.

Apoptosis or programmed cell death is a tightly regulated form of controlled cellular self-destruction. The induction of apoptosis in virus-infected cells is an important antiviral defense mechanism [34]. Some viruses have therefore evolved strategies to modulate host cell apoptosis in order to enhance their survival and dissemination [53]. CHIKV was shown to mobilize the apoptotic machinery of cultured fibroblasts to invade cell defenses [54]. We showed that ONNV strongly upregulated the proapoptotic factor TRAIL mRNA levels but also the anti-apoptotic Bcl 2 gene expression in infected SC. The capacity of ONNV to upregulate Bcl 2 gene expression may possibly prevent TRAIL-induced apoptosis in SC. Further studies are needed to investigate the mechanisms of apoptosis regulation in alphavirus-infected SC and their potential involvement in the pathogenesis of alphavirus-associated GBS.

Myelination is a process by which SC in the PNS produce the myelin sheath around the axonal membrane [12]. In peripheral nerve injury, SC play a major role in several aspects of nerve repair such as degeneration and remyelination [55]. SC originate from the embryonic neural crest. After birth, the SC retain their inherent plasticity which allows them to differentiate into non-myelinating and myelinating cells [56]. SC differentiation into mature myelinating cells is critical for remyelination and is under the control of several transcription factors including Egr2 [57]. Pathogen infection will potentially disturb these pathways, preventing SC from differentiating into cells able to support remyelination [14]. *Mycobacterium leprae* (*M. leprae*), a species of bacterium responsible for leprosy neuropathy in humans, can lead to PNS pathologies characterized by a process of nerve demyelination [58]. *M. leprae* was shown to infect and persist in SC and subsequent infection promoted dedifferentiation of host SC into progenitor stem-like cells with a marked downregulation of myelin genes (MPZ, MBP, and Erg2) and a reduced remyelination capacity in infected SC [58,59]. Myelin breakdown was shown to favor *M. leprae* viability and persistence inside SC [59].

Acute inflammatory demyelinating neuropathy may occur with CHIKV [60]. CHIKV can induce demyelination either directly by altering SC regenerative properties or through the immune inflammatory response to the virus and immune cells infiltration. In contrast to the capacity of *M. leprae* to downregulate myelin genes, we found that ONNV infection upregulated the expression of the pro-myelinating genes MPZ, MBP1, and the major pro-myelin transcription factor Egr2 in SC.

**Figure 12:**
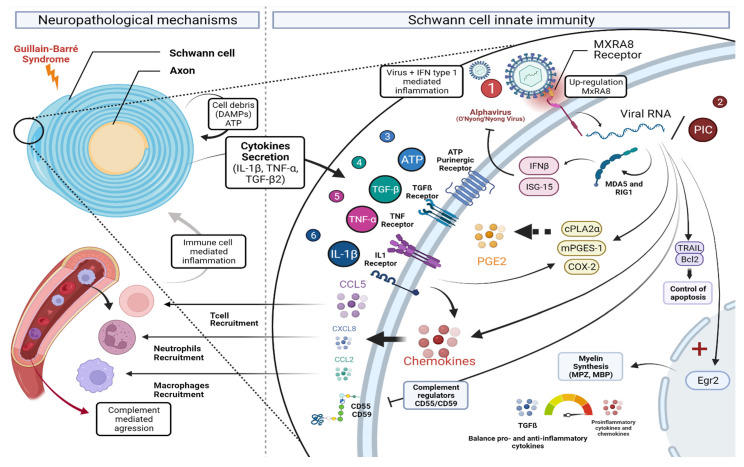
Summary figure: Primary infection of Schwann cells by alphaviruses and highlighting the different potential mechanisms of infective inflammatory neuropathies. Several mechanisms (yet ill-characterized) may be involved to instigate the inflammatory GBS-like response in peripheral nerves of patients infected by “old world alphaviruses” such as Chikungunya or O’Nyong Nyong Virus (ONNV). Canonically, it is well established that a pathogen can infiltrate a damaged blood peripheral nerve barrier and to cause bystander (indirect) injury to Schwann cells, demyelination, and axonal injury. Host cell debris (damage-associated molecular patterns DAMPs/alarmins such as ATP, HMGB1) may contribute further to the local inflammatory process. Indeed, the recruitment by chemokines (CCL2, CCL5, CXCL8) of innate (neutrophils, monocytes, and differentiating into macrophages) and adaptive (T lymphocytes) immune cells to the nerve will lead to the release of cytotoxic proinflammatory cytokines (IL1-β, TNF-α), outweighing those with anti-inflammatory activities (IL10, TGF-β). Other components such as prostaglandins (PGE2), complement, will stimulate altogether macrophages to phagocytose complement- or antibody-opsonized cell debris, including degraded myelin and axons. Critically, our data highlight herein, for the first time, a possible more direct mechanism of nerve injury after alphavirus infection. First, we describe that, in fact, SC expressing the MxRA8 alphavirus receptor ① can be infected directly by the prototype ONNV and yet capable of mounting a robust anti-viral response (IFN type I and ISG, e.g., ISG15) and as verified using the PIC viral analogue molecule ②. ONNV-infected SC were also mobilized in the robust production of all major inflammatory molecules listed above. We further observed that the infection led to upregulated expression of three key enzymes involved in the biosynthesis of PGE2 (cPLA2α, mPGES-1, and COX-2). The pro-inflammatory effects of ONNV infection (as tested at different MOI) were comparable (or even stronger) to that observed when SC were stimulated with recombinant cytokines or ATP ③ to ⑥. Interestingly, we also observed that ONNV-infected SC were able to upregulate several key factors associated with immunoregulation and tissue homeostasis. In response to infection, SC upregulated the expression of molecules involved in the control of apoptosis (e.g., Bcl 2) and, unexpectedly, the expression of myelin (transcription factor Egr2, Myelin P zero (MPZ), and MBP). Collectively, our original data suggest that GBS could potentially be mediated by a direct viral effect on SC. Alphaviruses can replicate within SC and induce a robust immunomodulatory response with potential importance in the GBS-like neuropathology. Figure created with Biorender.com (accessed on 5 May 2022).

In conclusion, our study demonstrated that SC, major PNS glial cells, are susceptible to alphavirus infection and that they are yet able to initiate robust antiviral and inflammatory responses following infection (summary Figure 12). This virus-mediated inflammation, with notably an important production of chemokines such as CCL5 by infected SC, can be potentially involved in the pathogenesis of PNS neurological complications, such as GBS. Our study provides new insights regarding the susceptibility and the remarkable immunomodulatory responses of SC to alphavirus infection of potential importance in the pathogenesis of GBS following alphavirus infection.

## Figures and Tables

**Table 1:** List of primers used for qRT-PCR.

Gene	Forward Sequence (5′-3′)	Reverse Sequence (5′-3′)
GAPDH	TGCGTCGCCAGCCGAG	AGTTAAAAGCAGCCCTGGTGA
CXCL8	CAGAGACAGCAGAGCACACA	GGCAAAACTGCACCTTCACA
CCL5	TCCTCATTGCTACTGCCCTC	TCGGGTGACAAAGACGACTG
CCL2	CTGCTCATAGCAGCCACCTT	CTTGAAGATCACAGCTTCTTTGGG
MxRA8	TTACTGTGGCCTGCACGAAC	CTCTCGGGGACGATGACATT
ONNV E2	CCCCTGACTACACGCTGATG	CCTTCATTGGAGCCGTCACA
ONNV NSP2	GCGGAGCAGGTAAAAACGTG	TAGAACACGCCCGTCGTATG
COX-2	TGGCTACAAAAGCTGGGAAG	GGGGATCAGGGATGAACTTT
cPLA2α	CATGCCCAGACCTACGATTT	CCCAATATGGCTACCACAGG
mPGES-1	CCAAGTGAGGCTGCGGAAGAA	GCTTCCCAGAGGATCTGCAGA
15-PGDH	TGCTTCAAAGCATGGCATAG	AACAAAGCCTGGACAAATGG
MPZ	GGCTGTGCTGCTCTTCTCTT	CCACTCACTGGACCAGAAGG
RIG-I	GCTATCGGGTCAACAACAGCTT	CCATATCTCAGCTGGGTGACAAA
MDA5	CTGTTTACATTGCCAAGGATC	ACACCAGCATCTTCTCCATTT
IFN-β	GTCACTGTGCCTGGACCATA	ACAGCATCTGCTGGTTGAAGA
ISG15	AGATCACCCAGAAGATCGGC	GAGGTTCGTCGCATTTGTCC
Egr2	GGCCCCTTTGACCAGATGAA	TTCTAGGTGCAGAGACGGGA
MBP1	GGCAAGGTACCCTGGCTAAA	GGGTGGTGTGAGTCCTTGTA
CD55	GCAACCATCTCCTTCTCATGTAAC	GTGGTGGTGCTGGACAATAAATT
CD59	ACTGCAAAACAGCCGTCAAT	AGGATGTCCCACCATTTTCA
TRAIL	TGAAGCAGATGCAGGACAAGT	TGGTTTCCTCAGAGGTTCTCAAA
Bcl2	TTCCTGCATCTCATGCCAAG	CTGGGAGGAGAAGATGCCC

## Data Availability

All data are fully available.

## Supplementary Materials

The following supporting information can be downloaded at: https://www.mdpi.com/2073-4409/12/1/100/s1, Figure S1: The infection and expression of high levels of capsid viral protein following CHIKV infection and comparing Schwann cells together with fibroblast-like synoviocytes; Figure S2: ONNV induces SC cytotoxicity at 48 h; Figure S3: ONNV does not increase CCL2 and CXCL8 proinflammatory chemokine production at 30 h post-infection; Figure S4: ONNV infection enhances the PGE2- synthesizing enzyme PGES detection in SC; Figure S5: SC modulate prostaglandin E2- biosynthesis pathway in response to the proinflammatory cytokines IL-1β and TNF-α stimulations.
