# Supplementary material for: Deciphering the Role of Schwann Cells in Inflammatory Peripheral Neuropathies Post Alphavirus Infection
Source: Cells. 2022 Dec 26;12(1):100. doi: 10.3390/cells12010100 (PMC9916230; doi:10.3390/cells12010100)
Supplement: Supplementary file 1 [file cells-12-00100-s001.zip › cells-1842806-supplementary.pdf]

Supporting information legends:

Figure S1. The infection and expression of high levels of capsid viral protein following CHIKV infection and comparing Schwann cells together with fibroblast-like synoviocytes. Cells cultured on coverslips were either mock infected or infected with different multiplicity of infection (MOI 10^{-1} to 10^{-4}) for 24h. After several washes in PBS, cells were fixed with ethanol (90%) prior to immunostaining with rabbit anti-capsid followed by secondary Donkey anti-Rabbit conjugated to Alexa 594 (Red). Nuclei were counterstained with DAPI (blue). Fluorescence imaging was carried out using Nikon fluorescence microscope. (mag x 200)

Figure S2. ONNV induces SC cytotoxicity at 48 h. SC were infected with ONNV (from MOI 10^{-3} to 1) or exposed to stimulatory treatments (PIC 10 µg/mL, IL-1β 10 ng/mL, TNF-α 10 ng/mL, TGF-β2 10 ng/mL and ATP 1 mM) for 48 h. Cytotoxicity was monitored by measuring percentage of LDH released in culture supernatant. Results are from 3 independent experiments. *: p-values ≤ 0.05, **: p-values ≤ 0.01 and ***: p-values ≤ 0.001 represent significant difference from controls (CT) by one-way ANOVA followed by the Bonferroni's test analysis.

Figure S3. ONNV does not increase CCL2 and CXCL8 proinflammatory chemokine production at 30 h post-infection. SC were infected with ONNV (from MOI 10^{-3} to 1) or exposed to PIC 10 µg/mL. Supernatants were harvested after 30 h and levels of CCL2 and CXCL8 were measured by ELISA assay. All experiments were done in triplicates and results are expressed as mean ± standard error. *: p-values ≤ 0.05, **: p-values ≤ 0.01, ***: p-values ≤ 0.001 and ****: p-values ≤ 0.0001 represent significant difference from controls by one-way ANOVA followed by the Bonferroni's test analysis.

Figure S4. ONNV infection enhances the PGE2- synthesizing enzyme PGES detection in SC. SC were cultured on coverslips, incubated with ONNV (MOI 1) for 24 h or mock infected (CT) and analyzed by fluorescence microscopy. Immunostaining using rabbit anti-CHIKV capsid and Alexa Fluor 594 (red)-conjugated donkey anti-rabbit or Alexa Fluor 488(green)-conjugated mouse anti-PGES. Nuclei were counterstained with DAPI (blue).

Figure S5. SC modulate prostaglandin E2- biosynthesis pathway in response to the proinflammatory cytokines IL-1β and TNF-α stimulations. cPLA2α (a), mPGES-1 (b), COX-2 (c) and 15-PGDH (d) mRNA levels were evaluated by qRT-PCR in SC exposed to stimulatory treatments (IL-1β 10 ng/mL, TNF-α 10 ng/mL, TGF-β2 10 ng/mL and ATP 1 mM) for 6 h and 24 h. All experiments were done in triplicates. Results are expressed as mean ± SEM and presented as normalized fold increase vs control. *: p-values ≤ 0.05, **: p-values ≤ 0.01 and ***: p-values ≤ 0.001 represent significant difference from controls by one-way ANOVA followed by the Bonferroni's test analysis.
