# Supplementary material for: Deciphering the Role of Schwann Cells in Inflammatory Peripheral Neuropathies Post Alphavirus Infection
Source: Cells. 2022 Dec 26;12(1):100. doi: 10.3390/cells12010100 (PMC9916230; doi:10.3390/cells12010100)
Supplement: Supplementary file 1 [file cells-12-00100-s001.zip › Figure S1 oct2022.pdf]

Primary human Schwann cells p5:

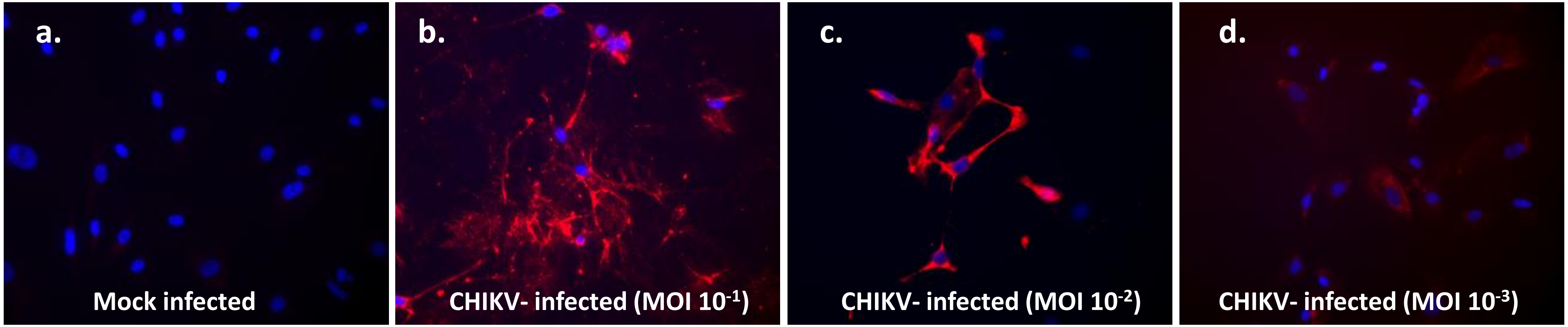

Primary human Fibroblast-like Synoviocytes p4:

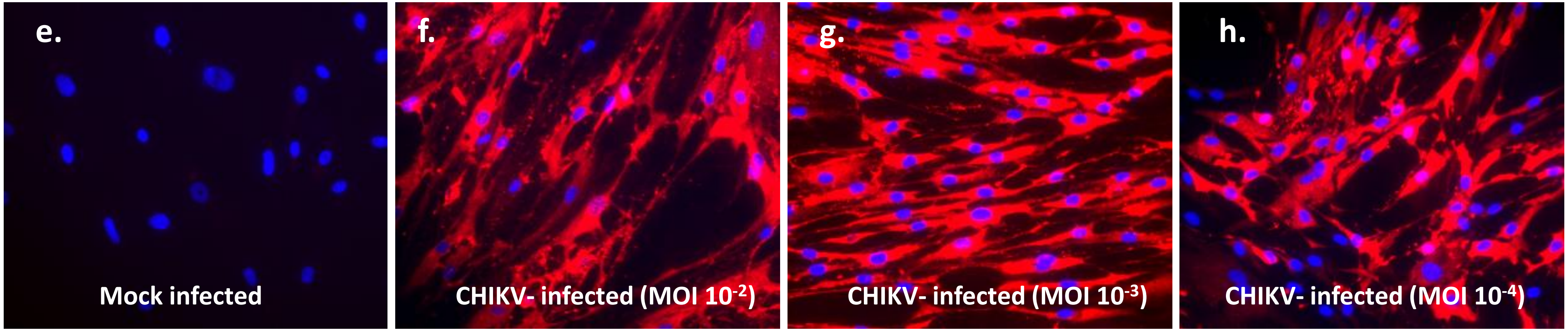

- **Supplemental figure S1**: Cells cultured on coverslips were either mockinfected or infected with different multiplicity of infection (MOI 10^{-1} to 10^{-4}) for 24h. After several washes in PBS, cells were fixed with ethanol (90%) prior to immunostaining with rabbit anti-capsid followed by secondary Donkey anti-Rabbit conjugated to Alexa 594 (Red). Nuclei were counterstained with DAPI (blue). Fluorescence imaging was carried out using Nikon fluorescence microscope. (mag x 200)
