# Supplementary figures and images for: Deciphering the Role of Schwann Cells in Inflammatory Peripheral Neuropathies Post Alphavirus Infection
Source: Cells. 2022 Dec 26;12(1):100. doi: 10.3390/cells12010100 (PMC9916230; doi:10.3390/cells12010100)

Figure S2

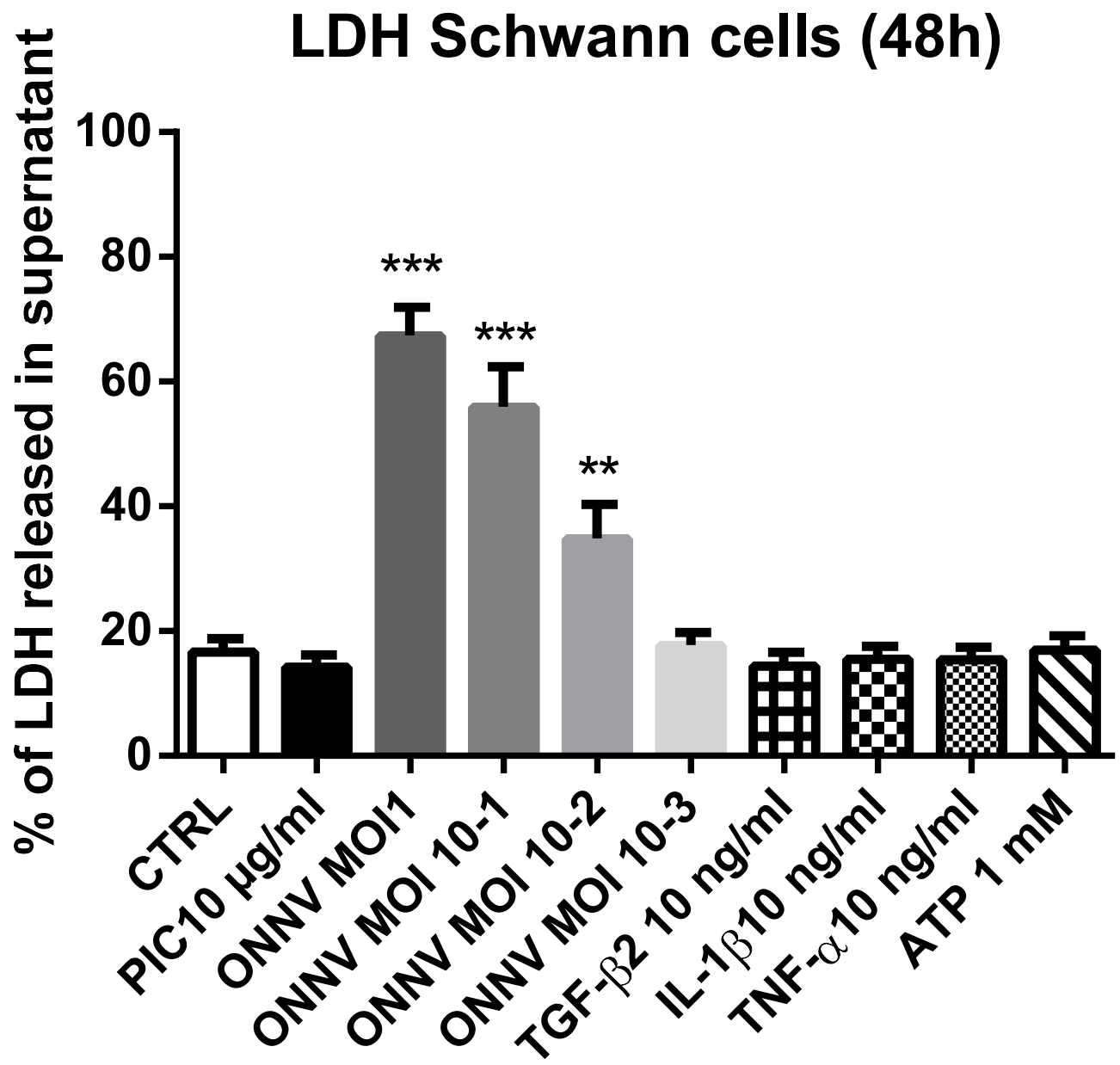

Supplement: Supplementary file 1 [file cells-12-00100-s001.zip › Figure S2 oct2022.pdf]

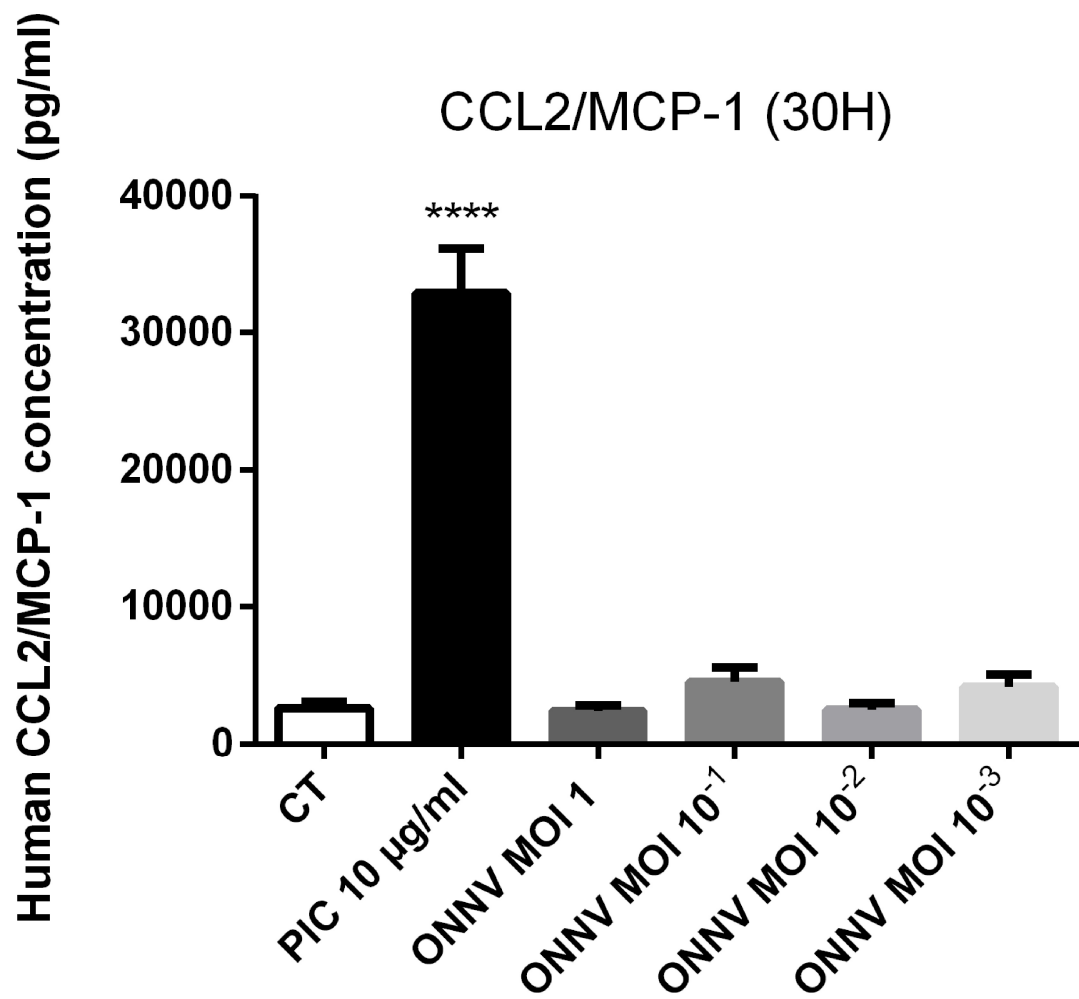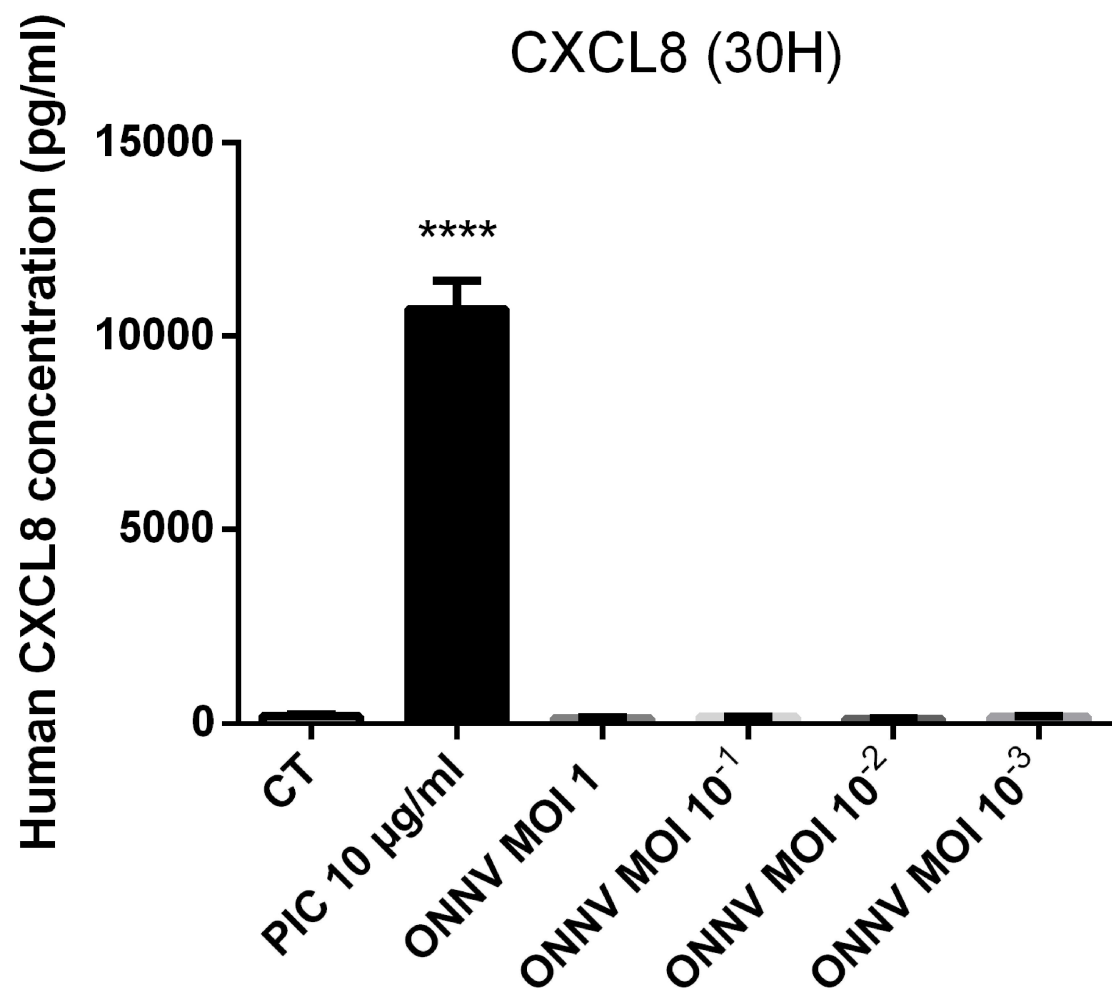

Supplement: Supplementary file 1 [file cells-12-00100-s001.zip › Figure S3 oct2022.pdf]

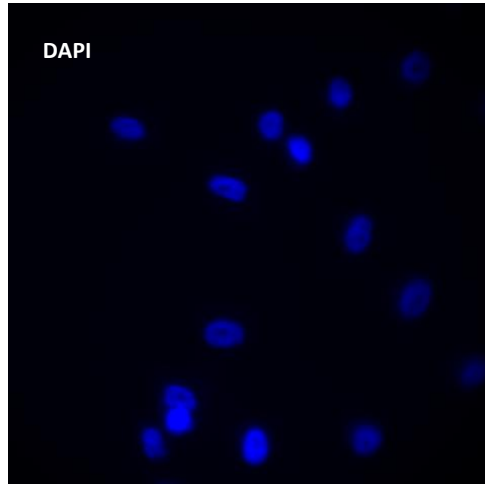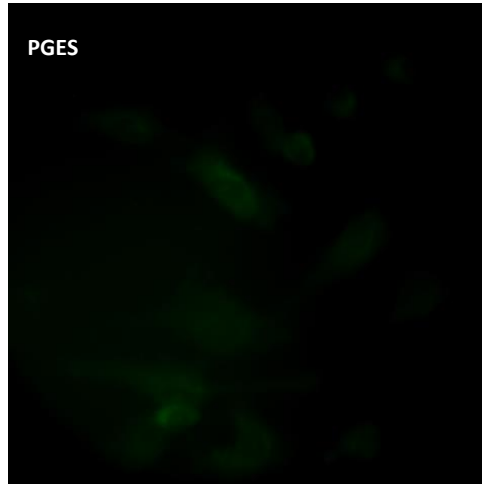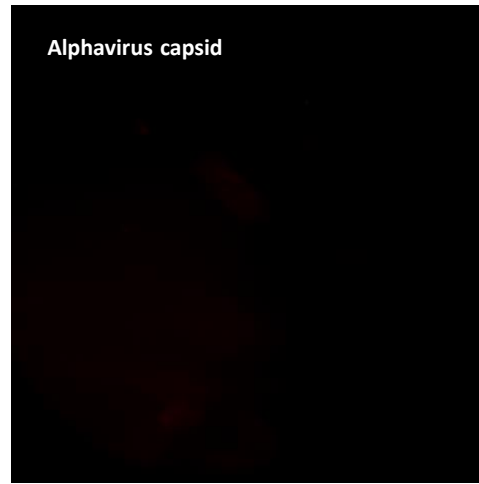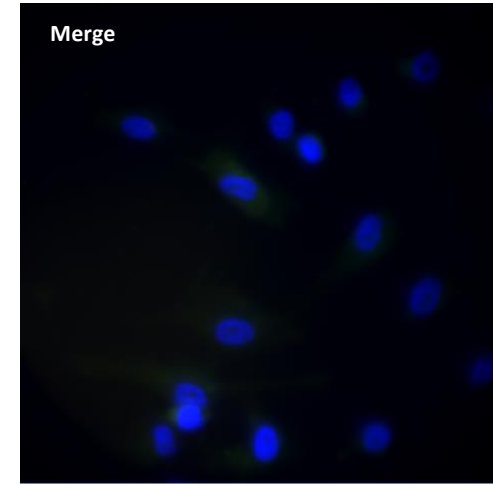

CT

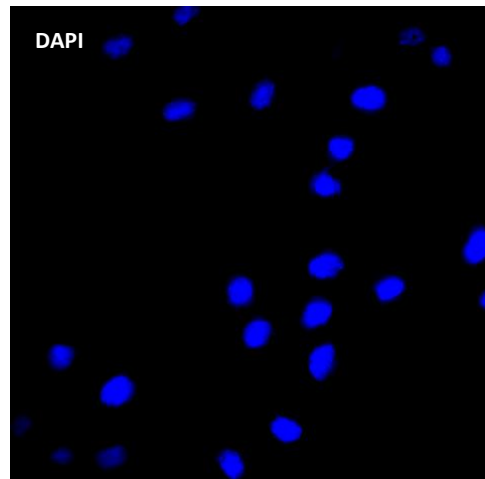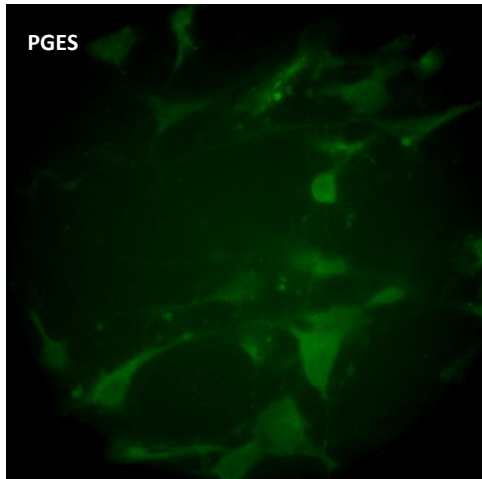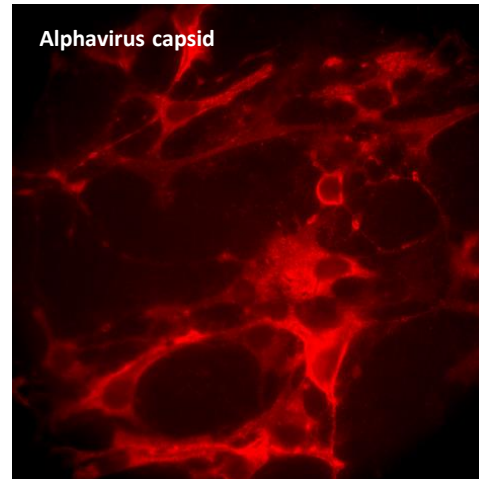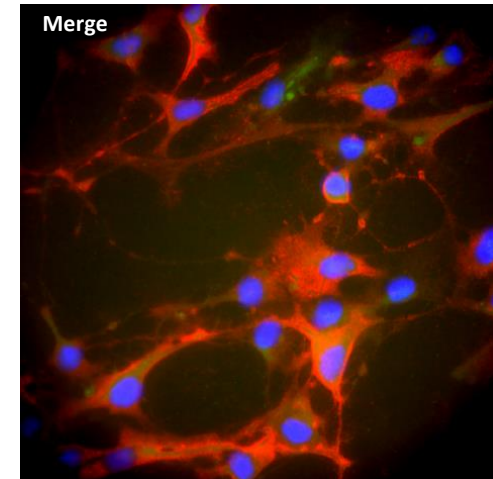

ONNV  
MOI 1

Supplement: Supplementary file 1 [file cells-12-00100-s001.zip › Figure S4 oct2022.pdf]

# Figure S5

a.

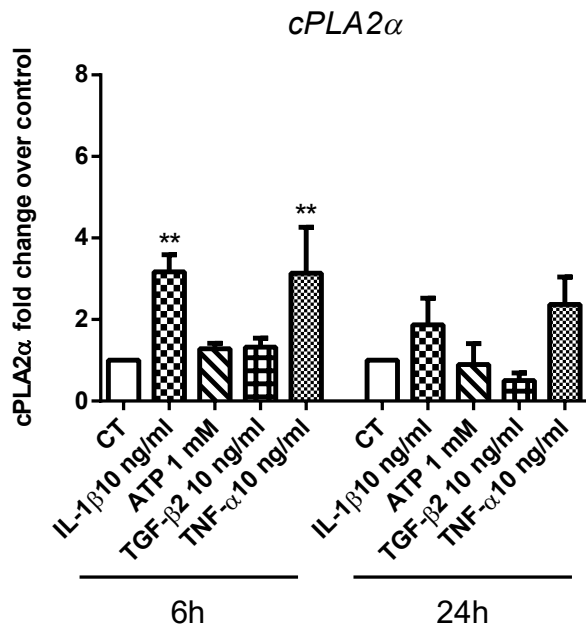

b.

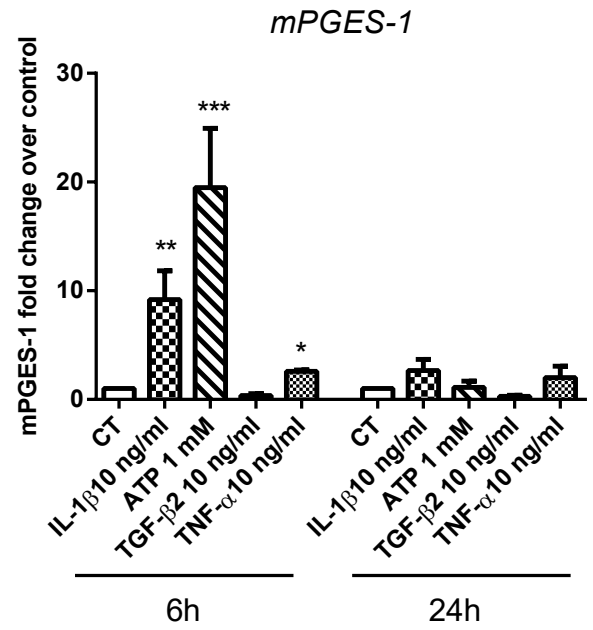

c.

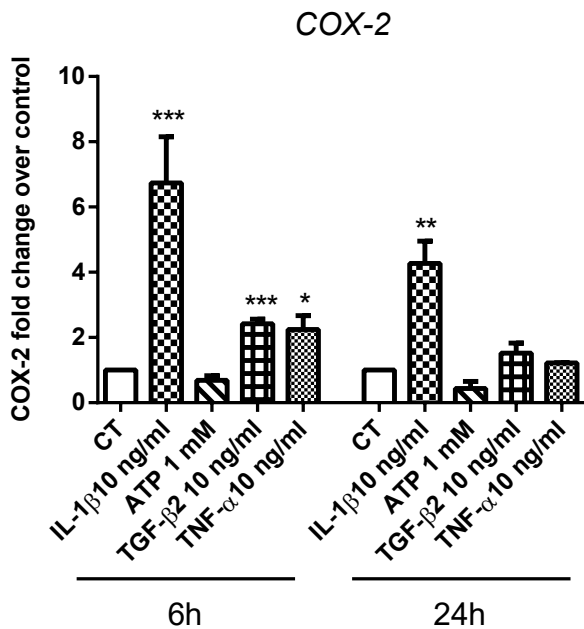

d.

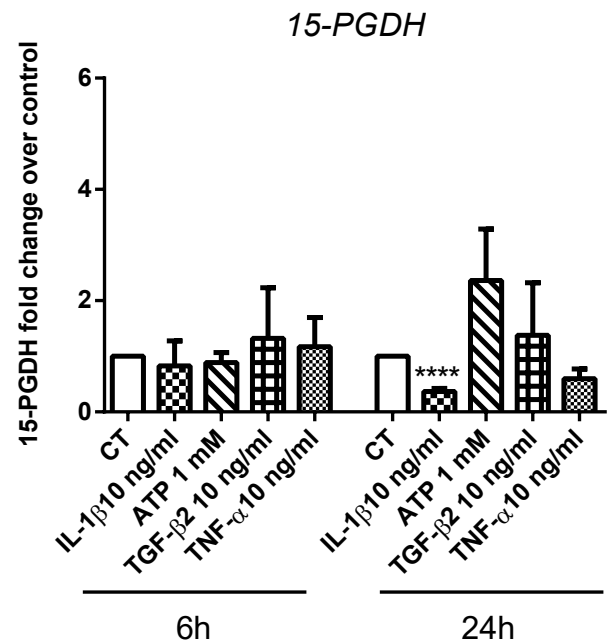

Supplement: Supplementary file 1 [file cells-12-00100-s001.zip › Figure S5 oct2022.pdf]
